# Supplementary material for: Extensive variation between chromosomes of North American and European hop
Source: Nat Commun. 2026 May 27;17:4110. doi: 10.1038/s41467-026-72379-8 (PMC13216280; doi:10.1038/s41467-026-72379-8)
Supplement: Supplementary file 1 — Supplementary Information [file 41467_2026_72379_MOESM1_ESM.docx]

**Extensive variation between chromosomes of North American and European hop**

Kale *et al.*

**Supplementary Note 1. Transcriptome and metabolome analysis of the developing hop cone**

Plants comprise an incomparably diverse multidimensional chemical space originating from highly complex biosynthetic processes. This is the fundamental basis that makes their sessile lifestyle possible. A key element is the production of diverse families of specialized metabolites that guide the plant to adapt to biotic as well as abiotic stresses in their environment e.g. defense against herbivores or attraction of pollinators^1^. Specialized metabolites are small molecules (< 1500 Da) and include a multitude of different structural classes: polyketides, terpenoids, N-containing compounds e.g. alkaloids and cyanogenic glucosides, and phenylpropanoids e.g. flavonoids. Throughout human history, plant specialized metabolites have been used as flavors, fragrances, pigments, medicines and industrial raw materials^2,3^. In the context of hops, their metabolites are utilized to improve the quality of beer by providing bitterness and aromatic flavors. This makes these constituents high-value bioactive products^4^. However, to harness the full biochemical potential of the chemical space produced by hop cones, it is crucial to understand the interplay of genetics and metabolism. In this study, a thorough metabolomic as well as transcriptomic analysis was conducted to describe the chemo-transcriptional framework of hop cone development in cv. Apollo.

To highlight the localization of key metabolites in cv. Apollo, such as bitter acids and the prenylated flavonoid xanthohumol, targeted metabolomics analyses based on Matrix Assisted Laser Desorption Ionization-Mass Spectrometry Imaging (MALDI-MSI) was conducted on cones collected 2 weeks after flowering (WAF). As expected, the localization of general metabolites as monitored by MALDI-MSI analyses (Supplementary Fig. 28 b) was restricted to the green tissues strig, bracts and bracteoles. the presence of α- and β-acids, the chalcone xanthohumol and the flavanone isomers 6-/8-prenylnaringenin (Supplementary Fig. 28 c) was detected in most of the imaged area. However, these compounds display an obvious much higher intensity outside the green tissues (brighter colours = higher intensity as indicated by the intensity bars next to the images), suggesting the synthesis of bitter acids as well as relevant prenylated phenylpropanoids to be localized to the lupulin glands. Therefore, the smear-like localization of these compounds is likely caused by delocalization and disruption of glands during sample preparation. To increase the intactness of the delicate bracts and bracteoles during sectioning, the viscous embedding medium carboxymethyl cellulose (CMC) was distributed between the tightly overlaid green tissues using a syringe that may have caused the glands to dislodge from the epidermal layer. Further, as this procedure could only be performed using fresh cones, they were subsequently frozen in the CMC at −78.5 °C^5,6^, i.e. slightly slower than snap freezing, possibly causing disruption of some glands in the process. Interestingly, the highest intensity areas of the bitter acids humulone and lupulone did not fully align with xanthohumol and 6-/8-prenylnaringenin, indicating different sites of biosynthesis and/or accumulation of these compounds.

Untargeted metabolomics was utilized to shed light upon the yet mostly undescribed chemical constituents produced in the course of hop cone development. In plants, specialized metabolism is often allocated to specialized tissues, such as glandular trichomes, that produce a wide range of structural diverse metabolites^7,8^. Accordingly, and based on the additional evidence from MALDI-MSI analysis, bract and glandular tissues derived from cv. Apollo were separately collected over five developmental stages, as also conducted in a previous study^9^. Subsequently, the volatile (VC) and non-volatile compound (NVC) profiles were analysed by employing high-resolution mass spectrometry analyses to maximize the spectral output inherent in the two tissues. This resulted in the isolation of 49 VC and 987 NVC, the latter group being represented by 424 and 563 constituents detected using negative and positive ionization modes, respectively. For a deeper understanding of the chemical diversity present in the non-volatile chemical space, a comprehensive computational dereplication pipeline that harnesses state-of-the-art molecular networking technology was applied. This approach constructs a spectral similarity network, based on the assumption that structurally related compounds have similar fragmentation patterns^10^. Thereby, individual chemical features are allocated by structural relatedness, forming subnetworks that represent chemical families. The network is then subjected to thorough chemical dereplication based on reference spectral data at three levels of confidence^11^, to identify already described compounds and subsequently highlight the presence of structural analogues in their spectral neighbourhood to ultimately provide a global chemical classification of the entire network^12^. The generated global molecular network of NVC in the course of hop cone development was dominated by lipid-related compounds (35%) (Supplementary Fig. 29), mostly due to the presence of a high number of terpenes and highly prenylated metabolites, such as bitter acids^13^. In addition, organic oxygen containing compounds (8%) and phenylpropanoids plus polyketides (7.5%) were identified as major chemical classes, underlining the role of polyphenolic compounds in hop cones. Level 1 and 2 metabolite identification was achieved for 37 VC and 103 NVC, including seven bitter acid analogs that could be identified with an in-house spectral library (humulinone, co-, ad- and n-humulone as well as co-, ad- and n-lupulone).

To investigate chemical intersections among contrasting cone tissues and developmental stages, hierarchical cluster analysis was conducted based on the combined set of 49 VC and 987 NVC. The generated chemical dendrogram displays metabolic adjacency among the sample set, with an evident subcluster emerging from the glandular fraction of stage 3 to 5, which suggests a distinct metabolite profile compared to the remaining samples (Supplementary Fig. 31). This chemical diversification in the glandular fraction begins already at stage 2, while glands of stage 1 together with the entire bract fraction form another subcluster, indicating a common metabolome. The metabolic cluster analysis displays no major chemical specialization within the bract fraction, as was found in the glands during cone maturation. These results highlight the relevance of the glandular fraction for specialized metabolite biosynthesis and diversification, as also indicated for bitter acids and prenylated phenylpropanoids using MALDI-MSI analysis (Supplementary Fig. 28).

The global molecular network further provides a spectral similarity map to study the undescribed chemical space around identified structural scaffolds. The chemical family of α-acids, which is predominantly inherent in the gland fraction, serves as an example. It contains 53 spectral features in total, including the described N-, Co- and Ad-humulone. Besides the latter, 47 putative chemical analogs (26 of negative and 21 of positive ionization origin) are present, highlighting a yet underexplored chemical space around the humulone scaffold. β-acids describe metabolites around the lupulone scaffold, which in this study form a chemical family of 20 spectral features, with 14 unknown analogs (5 of negative and 9 of positive ionization origin).

Our metabolite annotation revealed that approximately 90% of the non-volatile chemical space observed in hop cones could not be confidently identified through spectral matching with public spectral libraries - an outcome consistent with the nature of untargeted metabolomics^9^, which typically uncovers a substantial proportion of previously undescribed compounds and thus provides significant added value to exploratory studies. The chemical network presented in Supplementary Fig. 30 for example illustrates the extensive family of uncharacterized NVCs around the well-known brewing-relevant α-acids of the humulone type. These undescribed chemical features comprise ~89% of this chemical family, highlighting a plethora of structurally related yet unknown compounds. The potential biological relevance of these undescribed humulone analogs is suggested by their elevated abundance, compared to the identified N-, Ad- and Co-humulone (level 1 identification), as highlighted in Supplementary Fig. 32. In this context, it is noteworthy that the Cannabaceae specific PKS enzyme family, which includes the enzyme HlVPS that catalyzes the initial step in the biosynthesis of α- and β-acids in hops, also comprises several enzymes that are expressed in glandular trichomes but whose functions remain unknown. A phylogenetic analysis (Figure 5c) shows that this family is expanded in the Cannabaceae and the four enzymes with unknown function identified in hops are colocalizing on chr09 with the VPS and likely share a common origin. This serves as just one example of the many knowledge gaps that our genomic resource will help to address in the future. Particularly studies of the bitter acid biosynthetic pathway will benefit from the provided genome sequence, by supporting state-of-the-art multi-omics approaches with a highly resolved genetic resource crucial for accelerating our understanding of hop metabolism as well as bioengineering of valuable metabolites from hops^14^.

To augment our understanding of the biosynthetic machinery involved in the production of specialized metabolites during the development of hop cones, a transcriptomic analysis was conducted on cone material derived from cv. Apollo. For this, gene expression data generated from the glandular fractions that were collected over five developmental stages was utilized. After normalization across samples, gene expression data showed a high consistency in the distribution of expression values among the samples (Supplementary Fig. 34). Overall, 46977 genes of the total 61318 annotated genes in the Apollo genome were expressed in at least one stage. To highlight genes that show significant changes in expression over the course of cone development, a differential expression analysis was conducted. Herein, 3612 genes were found to be at least 4-fold differentially expressed at a significance of smaller or equal than 0.001 (FDR) in any of the pairwise sample comparisons. Based on these differentially expressed genes (DEG), a hierarchical cluster analysis was conducted. This generated four clusters with distinct gene expression trends throughout the five developmental stages studied (Figure 6b). All DEG clusters underwent quality control via a silhouette analysis that resulted in an average silhouette value of 0.69, while 137 DEG with negative values were removed from downstream analyses (Supplementary Fig. 35).

To understand the chemo-transcriptional relationships during hop cone development, a correlation analysis was done to point out DEG clusters associated with a distinct chemical profile (Figure 6c). Eventually, this will help to highlight links between aroma relevant chemistry and the genetic modules involved and serve to define how the observed chemical plasticity is regulated. For this, the NVC chemical space was refined to the 45 major chemical families, harbouring 59% of the observed chemical space (583 out of 987 features). Focusing on chemical families rather than single compounds is an essential step to guide the subsequent correlation analysis towards metabolic pathways^11^. In this analysis, an association of chemical and gene expression data was accounted for if at least 5% of DEGs of that tested cluster were found to have a Pearson coefficient above or equal to 0.9 (Supplementary Table 19).

The correlation of gene expression data to the combined metabolic profiles of 49 individual VC and 45 chemical families in the context of the developing hop cone, highlighted DEG clusters 2 and 4 as being associated with the majority of VC measured (29 out of 49 VC). In DEG cluster 2, these VC comprise flavour and aroma compounds like the monoterpenoid beta-myrcene, a range of fatty acyls and the corresponding esters. DEG cluster 4 is linked to a range of sesquiterpenoids, such as beta-caryophyllene and alpha-humulene, and additional fatty acid esters. Genes within cluster 2 are linked to a wide range of NVC, which relate to different terpenes, α and β-bitter acids as well as chalcones, such as xanthohumol, whereas DEG cluster 4 is solely linked to a NVC family of sesquiterpenoids, underlining the matching VC profile. NVC are dominating the correlated chemical space for DEG of cluster 3 and specifically linked to the presence of two terpene lactones, a diterpenoid and a sesquiterpenoid family around beta-caryophyllene oxide. Fifteen of these chemical families are shared with DEG cluster 2 that include α-bitter acids around humulone as well as a variety of terpene-related and organoheterocyclic compounds, indicating that the increased number of expressed genes within the initial three stages of cone development corresponds to the highly diverse chemical space. DEG cluster 1 aligns with the presence of a small selection of VC, comprising three mono- and two sesquiterpenoids including beta-ocimene and germacrene D, but does not correlate to any NVC family. This suggests that cone chemistry is not impacted by declining gene expression throughout cone development. Given the correlation of the majority of VC and NVC with the DEG belonging to cluster 2, the possible contribution of haplotype specific alleles to the expression of specialized metabolites of interest was examined. An initial screen for orthologous gene pairs revealed 194 homozygous and 380 heterozygous genes in this cluster (Supplementary Table 18). Only heterozygous genes can be assessed for ASE, and a phase-resolved differential expression analysis revealed 45 orthologous gene pairs with significant ASE (*P <* 0.05). Here, *valerophenone synthase* (*hlvps*) was found to have a preference towards phase 2 gene expression (Figure 6d).

To deepen the insight into ASE of annotated genes in the whole Apollo genome, homo- and heterozygous gene pairs were determined, resulting in 19725 orthologue pairs of genes. The entire hop cone developmental gene expression dataset was used to test for significant cases of ASE. This revealed 6178 heterozygous gene pairs with adjusted *P <* 0.05, while no homozygous genes were found significant. Accounting for significant and non-significant ASE on a genome-wide scale unveiled a mostly equal distribution of the proportion of expressed genes (Supplementary Figure 24). The only exceptions are chromosomes 3, 5 and 10 that show significant (*P* < 0.05) ASE towards phase 1 as documented by applying a two-proportions Z-test (Supplementary Table 20). For a stage-resolved insight into ASE during hop cone development, the gene expression set of the 19725 orthologous gene pairs was employed within a stage-separated differential expression analysis (Supplementary Figure 36). Stage 4 and 5 exhibit considerably more gene pairs that show significant ASE compared to the earlier stages, underlining the role of genetic plasticity in specialized metabolism during cone maturation as seen for DEG cluster 2.

**Supplementary Fig. 1*.* Distribution of read length of circular consensus sequence (CCS) reads generated using PacBio HiFi sequencing per batch.** A total of 120 Gb circular consensus sequence (CCS) reads with an average length of 22 kb corresponding to 24-fold coverage of the diploid genome of cultivar (cv.) Apollo were generated. Source data are provided as a Source Data file.


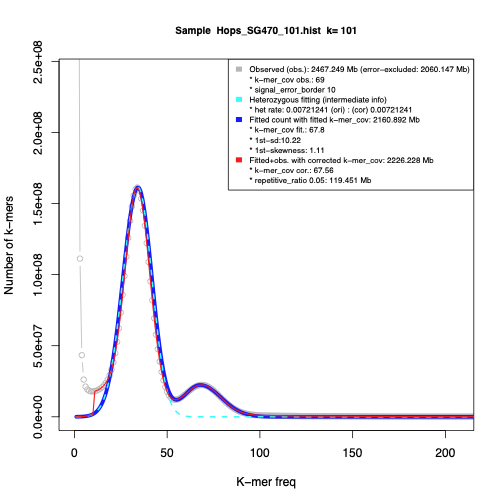


**Supplementary Fig. 2. k-mer based estimation of genome size of cv. Apollo.** The estimated genome size of cv. Apollo is 4.4 Gb. Source data are provided as a Source Data file.


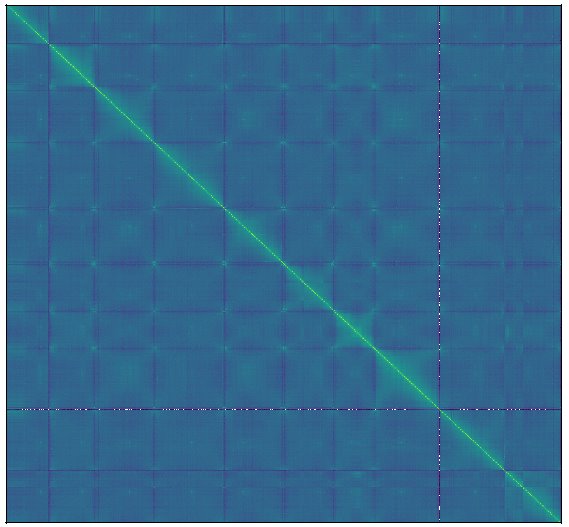


**Supplementary Fig. 3. Intra and inter chromosomal Hi-C contact matrices for the haploid assembly of cv. Apollo.** The chromosomes are renamed as per the *Cannabis sativa* genome and ordered serially from 1 (chr01) to 10 (chrX). The Hi-C contacts are visualized as heatmaps where rows and columns represent genomic regions and color intensity indicates interaction frequency. A strong signal intensity along the diagonal indicates high contact probability and contiguity of the pseudomolecules.


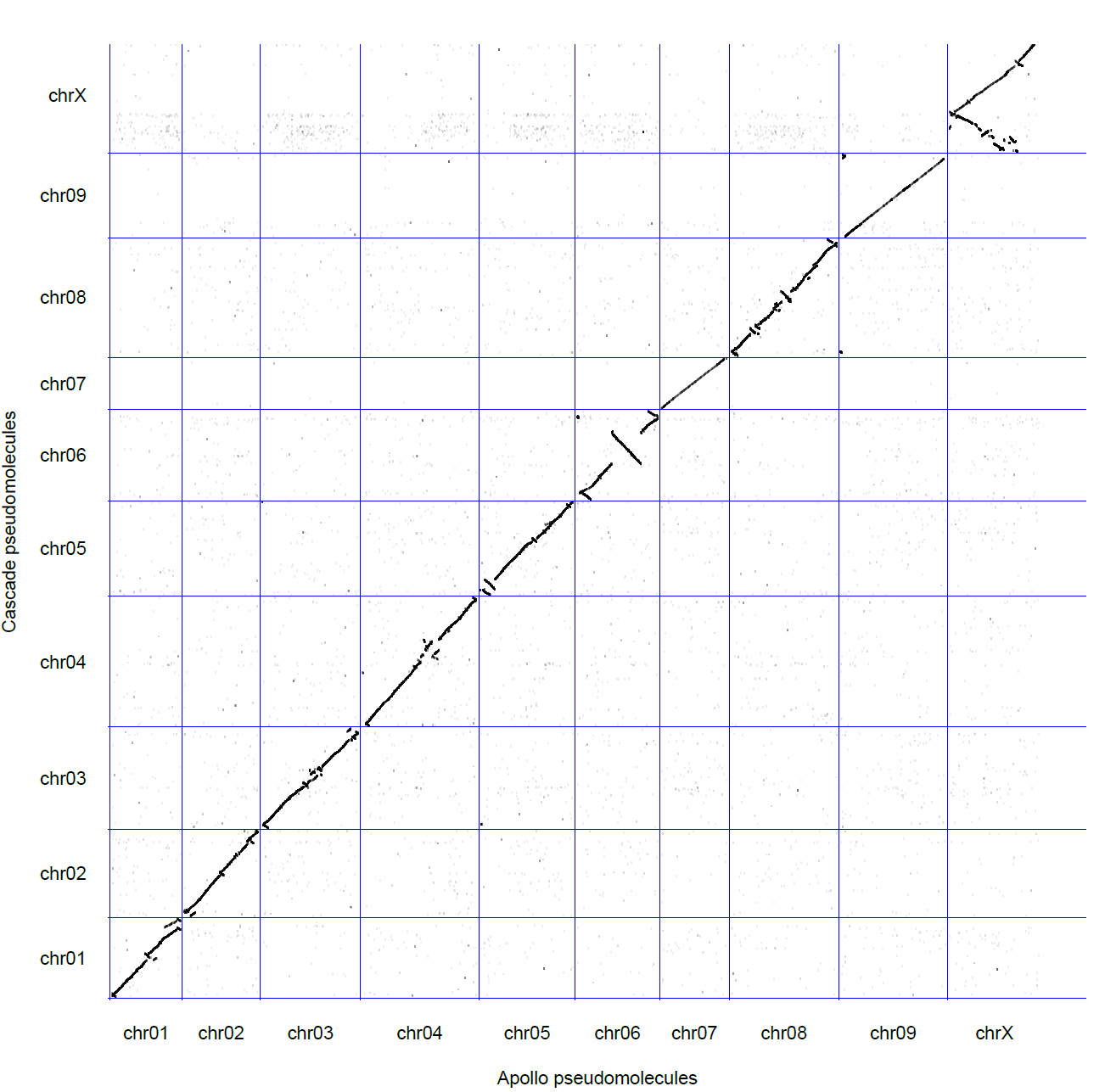


a

b

**Supplementary Fig. 4. *C*omparison of the haploid assembly *of Humulus* cv. Apollo to the *Cannabis sativa* Cs10 reference genome (GenBank: GCA_900626175.2).** a) Dot plot showing collinearity between the two available haploid *Humulus* assemblies of cvs. Apollo (HA, this study) and Cascade (HC). The collinearity was assessed by assembly-to-assembly comparison. Cascade pseudomolecules were aligned to the chromosomes of the haploid Apollo assembly. Primary alignments with length ≥ 2000 bp were extracted and visualized as a dot-plot revealing overall contiguity between the assemblies. The cv. Cascade assembly was shown to contain duplications interpreted as syntenic blocks within the haploid genome^15^. The results of this assembly-to-assembly comparison to Apollo however suggest the presence of remaining allelic contigs within the haploid Cascade assembly, resulting in the rather large assembly size of 3.71 Gb. b) A scaled bar plot illustrating proportion of gene models aligned against the haploid Apollo hop assembly. This reveals a one-to-one relationship for most of the chromosomes. The information was used to rename the *Humulus* chromosomes. Source data are provided as a Source Data file.

**Supplementary Fig. 5. Distribution of gene density across the pseudomolecules of the haploid cv. Apollo assembly.** The number of gene models in 1 Mb bins were calculated and plotted against their positions on the pseudomolecules. Source data are provided as a Source Data file.

**
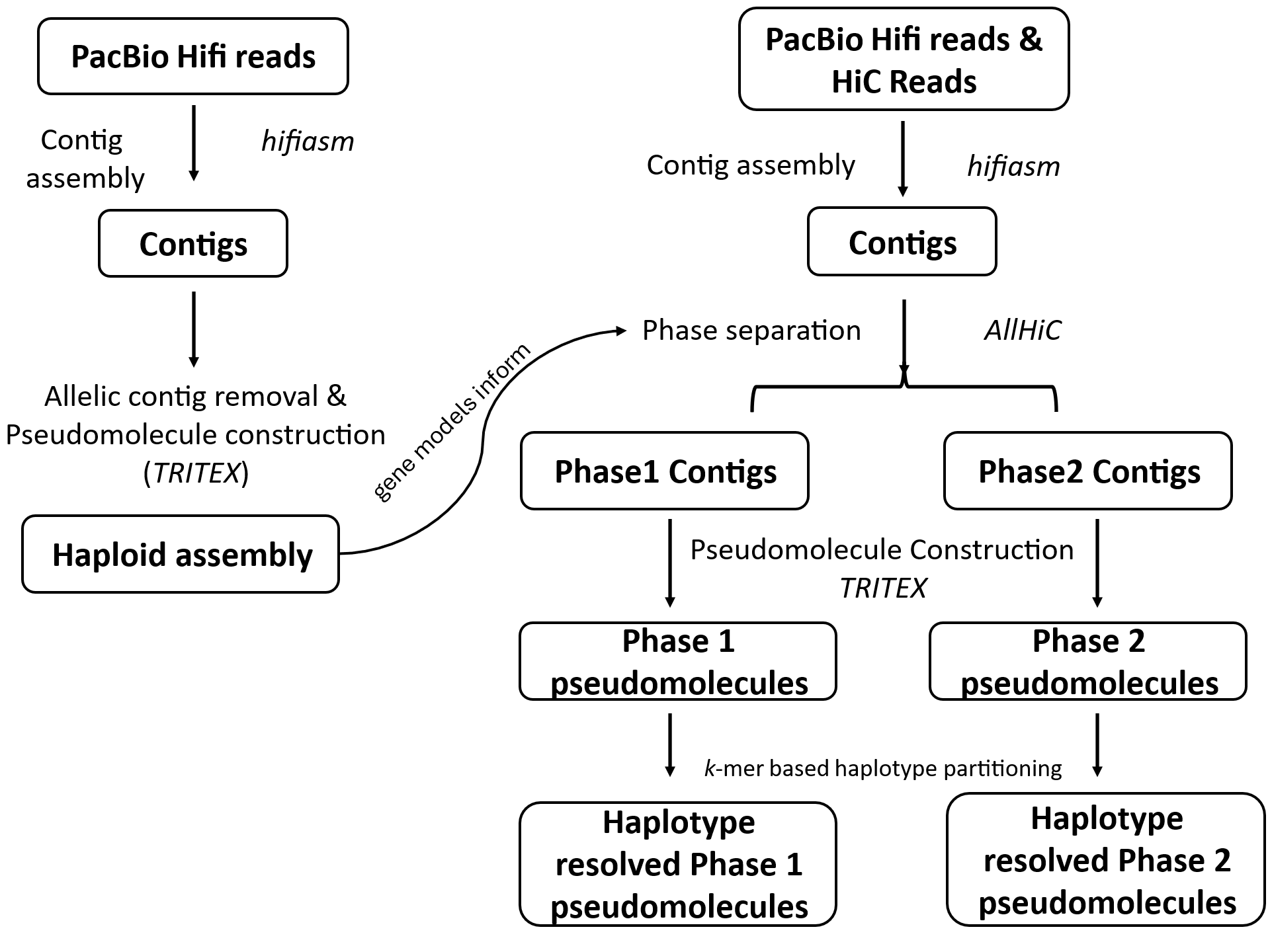
**

**Supplementary Fig. 6. Genome assembly workflow.** PacBio Hifi reads were assembled using *hifiasm* to generate a contig assembly (for assembly statistics see Supplementary Table 2). The primary contigs along with genetic map and Hi-C reads were used for pseudomolecule construction using TRITEX. The allelic contigs were removed by visualizing the Hi-C contact matrix heatmap. For the phased assembly, Hifi reads and Hi-C data were assembled into contigs using *hifiasm*, and contigs were partitioned into haplotypes with the help of ALLHiC. For this, the gene models from the haploid assembly were aligned to merged phased contigs and allelic contig pairs were identified. The Hi-C contact information was then used to separate allelic contigs into two haplotypes. The contigs from each haplotype were assembled into pseudomolecules using TRITEX pipeline. High-copy k-mers were subsequently used to assign pseudomolecules to their parental European or North American origin resulting in the final haplotype resolved phased assembly of cv. Apollo (for assembly statistics see Supplementary Table 5). K-mers were derived from phase-specific tandem repeat clusters identified with TandemRepeatFinder and clustered into families with *vmatch* as described in Methods.


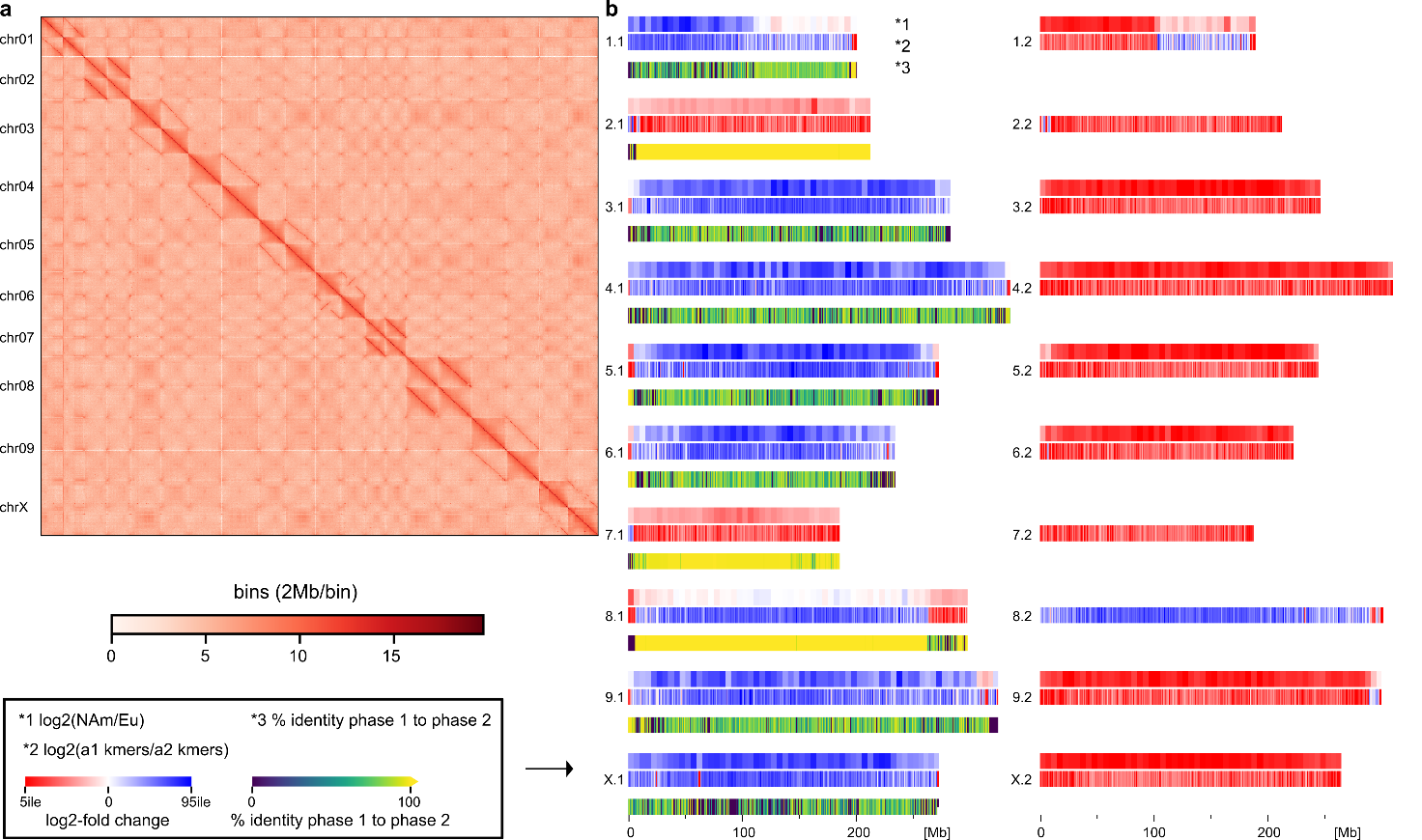


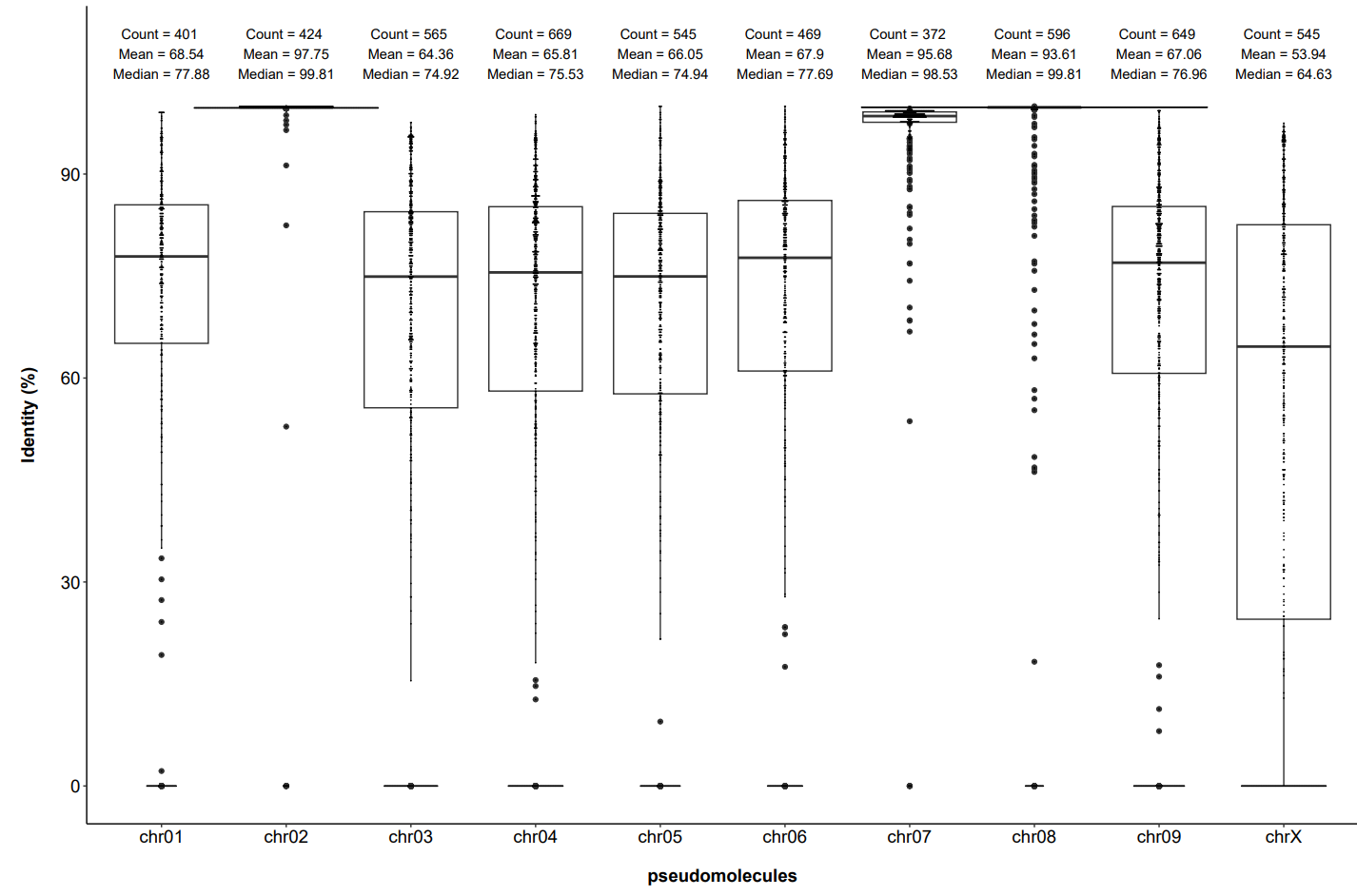


Supplementary Fig. 7. Overview Apollo assembly. a) Intra and inter chromosomal Hi-C contact matrices for the phased Apollo assembly. The Hi-C reads were mapped against the combined phased assembly and interaction frequencies are visualized as a heat map. The horizontal and vertical axes represent the chromosomes from phase resolved hop assemblies ordered as chr01.1, chr01.2, chr02.1, chr02.2, etc. Strong intensity signals along the diagonal indicate high contact probability, while strong lines parallel to the diagonal indicate inter-chromosomal collinearity and identity. b) Chromosomal distribution of ancestral origin indicators in the phased Apollo assembly. Top row: ratio of GBS read counts from North American (NAm, blue) and European (Eu, red) lines in 5 Mb bins as log_2_-fold value. Reads were mapped to a combined Apollo assembly, which contains only one copy of the homozygous chromosomes chr02, chr07 and chr08. Middle row: ratio of coverage with parent specific k-mers (blue vs red parent) in 0.5 Mb bins as log_2_-fold value. Parent specific tags were derived from discerned parental bins identified after plotting the chromosomal location of each of the largest 200 tandem repeat families, as few families showed a distinct opposite specificity for one or the other parent. Bottom row: for phase 1 chromosomes percent of sequence identity to phase 2 (yellow high identity, blue low) in 0.5 Mb bins. c) Chromosome wide distribution of percentage identity. Percentage of sequence identity (number of mismatches/lengths of alignment) × 100) was calculated for primary alignments with size ≥ 10 kb. Data are shown for each pseudomolecule as a box and whisker plots. In each plot, the centre line indicates the median, the lower and upper bounds of the box indicate the 25^th^ and 75^th^ percentiles, and the whiskers extend to the smallest and largest values within 1.5 times the interquartile range from the box. Points beyond the whiskers are shown as outliers. The total number of alignments (counts), together with the mean and median percentage identity for each chromosome, is indicated above each box. Source data are provided as a Source Data file.


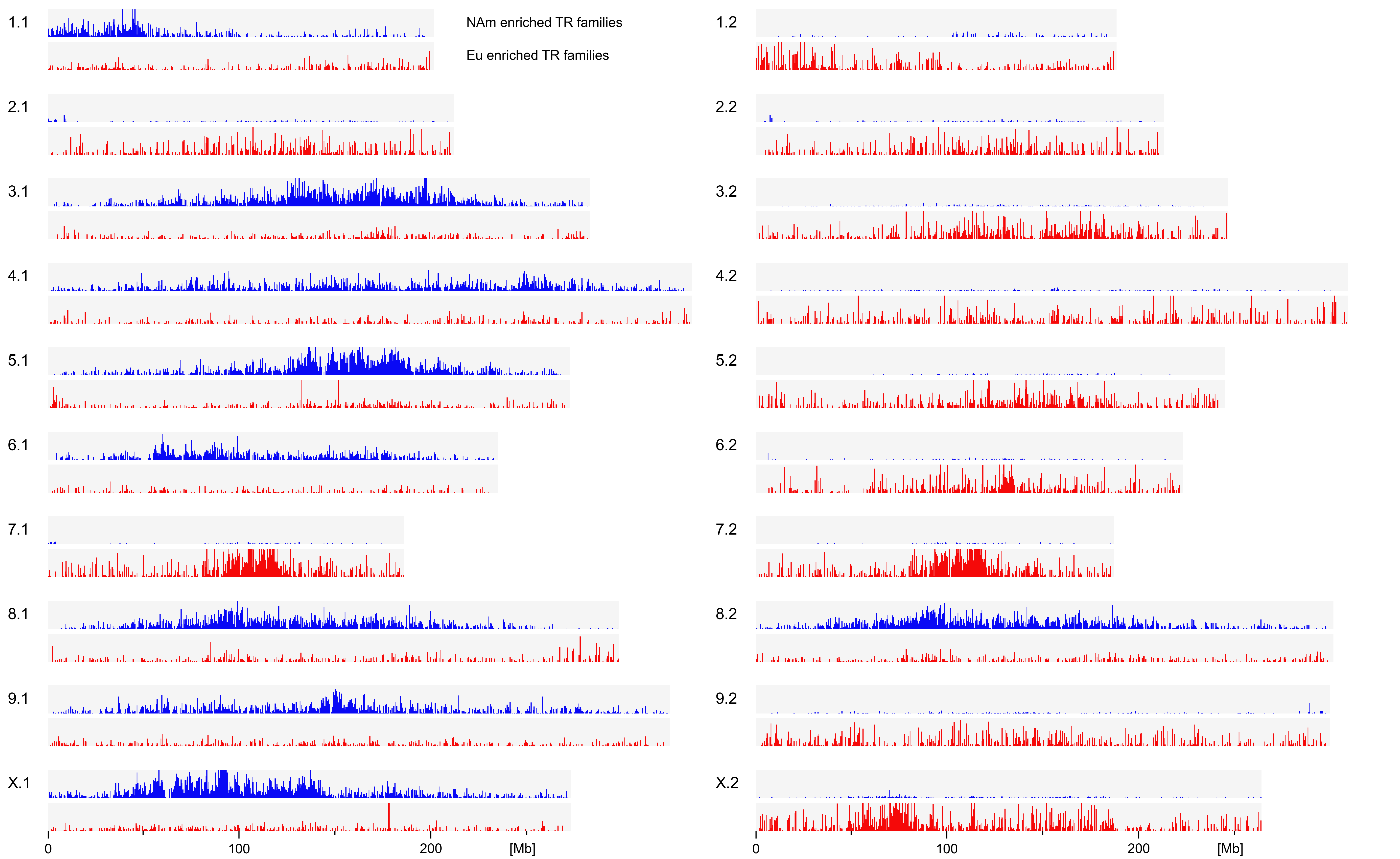


**Supplementary Fig. 8. Chromosomal distribution of parent specific tandem repeat (TR) families in the Apollo assembly per 0.5 Mb window.** The upper row per chromosome shows TR families enriched in the North American (NAm, blue) ancestry (*Y*_max_ = 1%), the lower row TR families enriched in the European (Eu, red) ancestry (*Y*_max_ = 0.3%). This initial rough binning was used to identify parent enriched sequence tags based on k-mers. Source data are provided as a Source Data file.


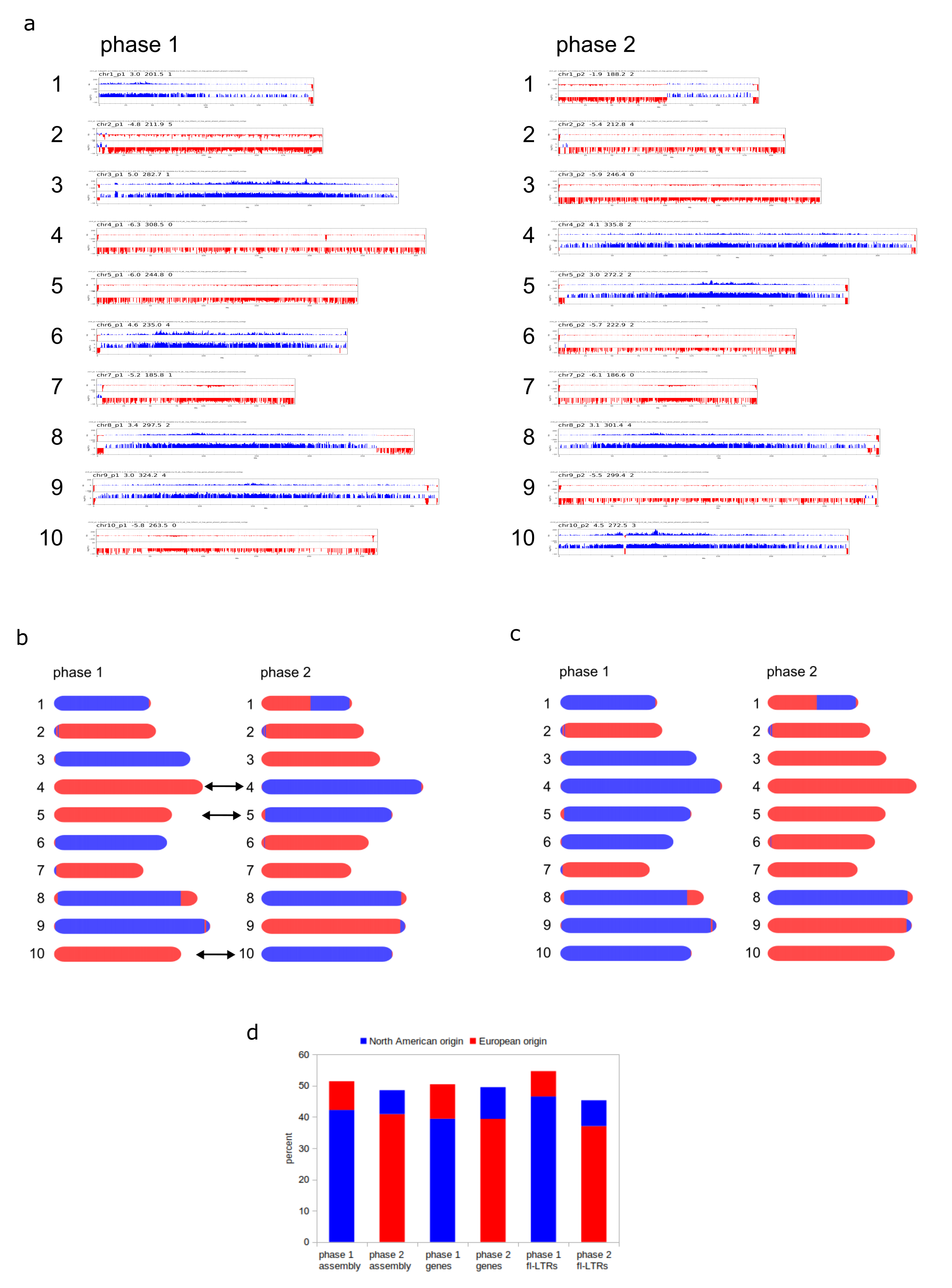


**Supplementary Fig. 9. Guiding the phase affiliation of Apollo pseudomolecules through parent specific sequence tags.** a) Initial phase 1 and phase 2 chromosome assignment from the phase agnostic assembly process. The upper row for each chromosome shows the absolute amounts of blue (North American) and red (European) parent sequence tags in kb per 0.5 Mb bin, the lower row the log_2_-fold ratio of blue/red per bin. b) Parental origin in the initial assembly. c) Parental origin after switching the phase affiliation for chromosomes 4, 5 and 10 in the final assembly to maximise parent homogeneity for each phase. d) Extent of parental admixture in phase 1 and phase 2 for assembly size, genes, and full length LTR-retrotransposons displayed as percentages. Source data are provided as a Source Data file.


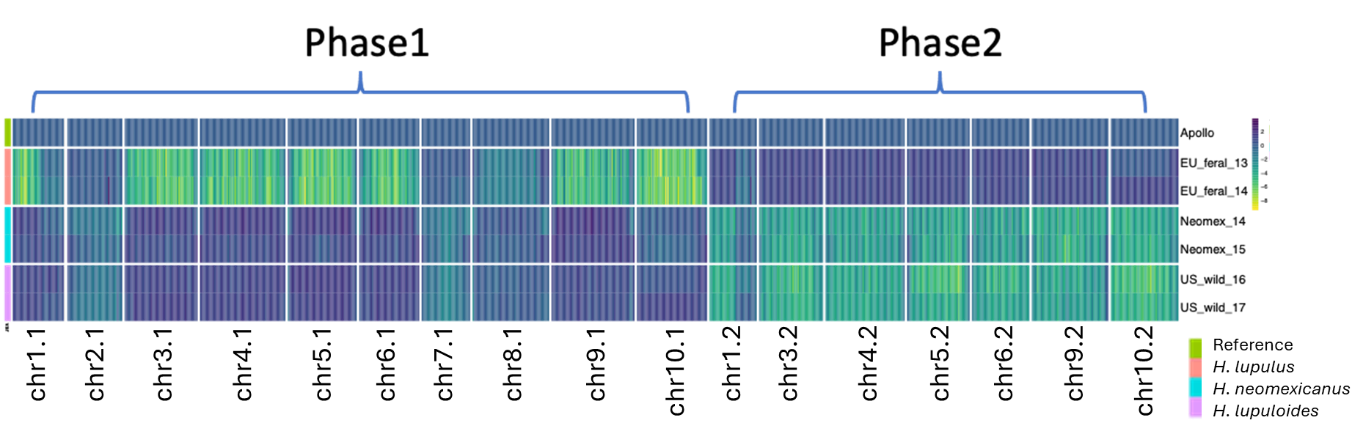


Supplementary Fig. 10. Heatmap of depth of coverage of selected accessions. A depth of coverage analysis was carried out to determine the origin of phases in cv. Apollo. For this, representative accessions from *H. lupulus*, *H. lupuloides*, and *H. neomexicanus*, were sequenced using genotyping-by-sequencing (GBS). As the phases of chr02, chr07 and chr08 were highly similar, we designed a special assembly by selecting all the chromosomes from phase1 and all but chr02, chr07 and chr08 chromosomes from phase2 of Apollo genome for read mapping. The number of uniquely mapped reads in 1mb bin were calculated and normalised by total number of uniquely mapped reads from that accession. Further, in order to compare the coverage with cv. Apollo, a second round of normalisation was carried out by dividing the normalised value for 1 mb window in an accession with the normalised value for the same 1 Mb window from Apollo. Higher depth of coverage was identified on phase 2 in accessions from Europe (*H. lupulus*). In contrast, accessions from North America showed higher depth of coverage on chromosomes from phase1. This indicates that phase1 in Apollo is inherited from North America while phase2 is inherited from Europe. Source data are provided as a Source Data file


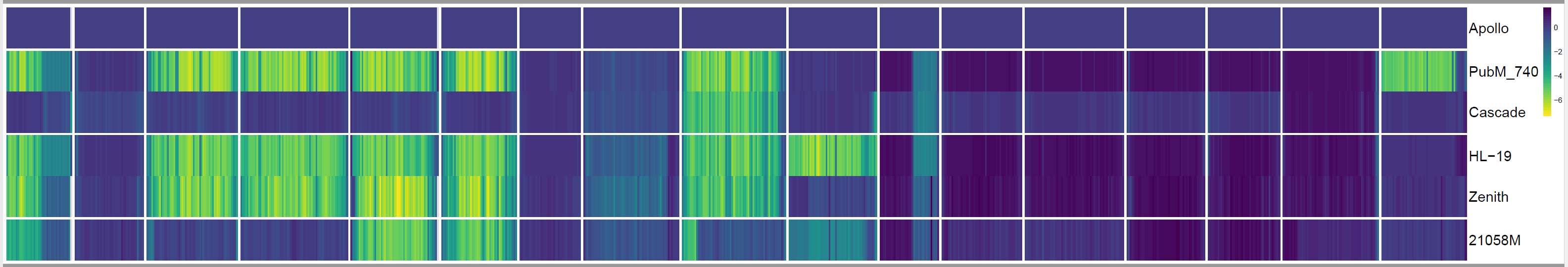


Supplementary Fig. 11. Assignment of North American and European ancestry to the chromosomes of Cascade, HL-19-060-002M, PubM_740, Zenith and USDA21058M. The chromosome origins were determined based on the depth of coverage analysis using genotyping-by-sequencing (GBS) reads of each accession. The log_2_(normalized reads) in 5 Mb window were plotted as a heat map. The chromosomes are separated by blank lines. The 10 columns from left represent 10 chromosomes from phase 1 while the remaining 7 columns represent chr01, chr03, chr04, chr05, chr06, chr09, and chrX from phase 2 of the Apollo assembly. The positive value indicates higher read coverage while negative values suggest low coverage because of absence of chromosome phase in respective samples. Source data are provided as a Source Data file.


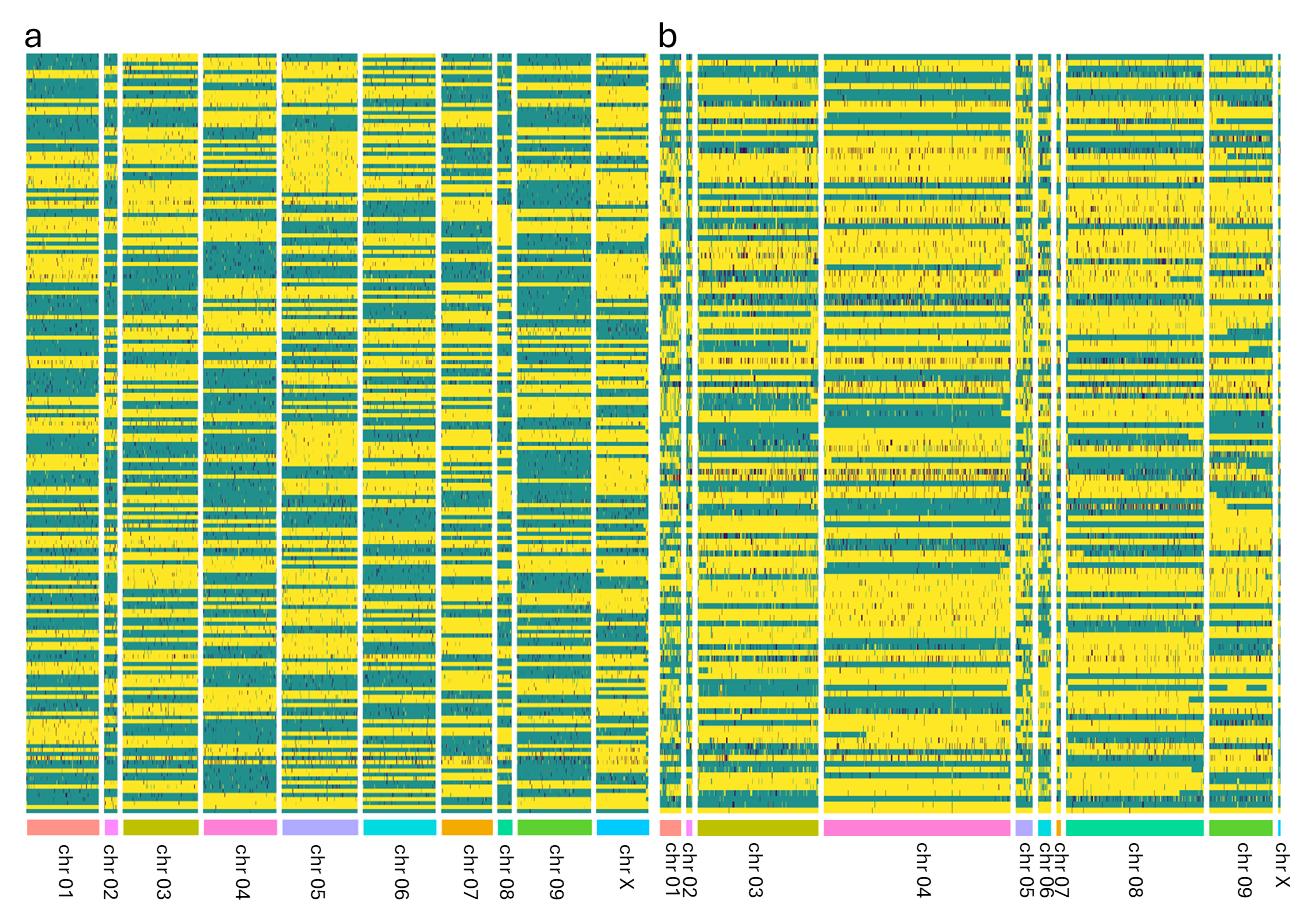


Supplementary Fig. 12. Biparental mapping showing suppressed recombination in cvs. Apollo and Zenith. Graphical genotypes of 1:1 segregating markers that are a) heterozygous in Apollo and homozygous in PubM_740 in Apollo × PubM_740 population or b) heterozygous in Zenith and homozygous in USDA21058M in the Zenith × USDA21058M population. Each row represents individual progeny while each column represents single SNP. The chromosomes are separated by vertical empty line. Heterozygous SNPs are highlighted with green colour; homozygous SNPs are highlighted with yellow colour while purple colour shows missing information. Continuous yellow or green rows indicates lack of recombination within respective progeny of a population. Source data are provided as a Source Data file.


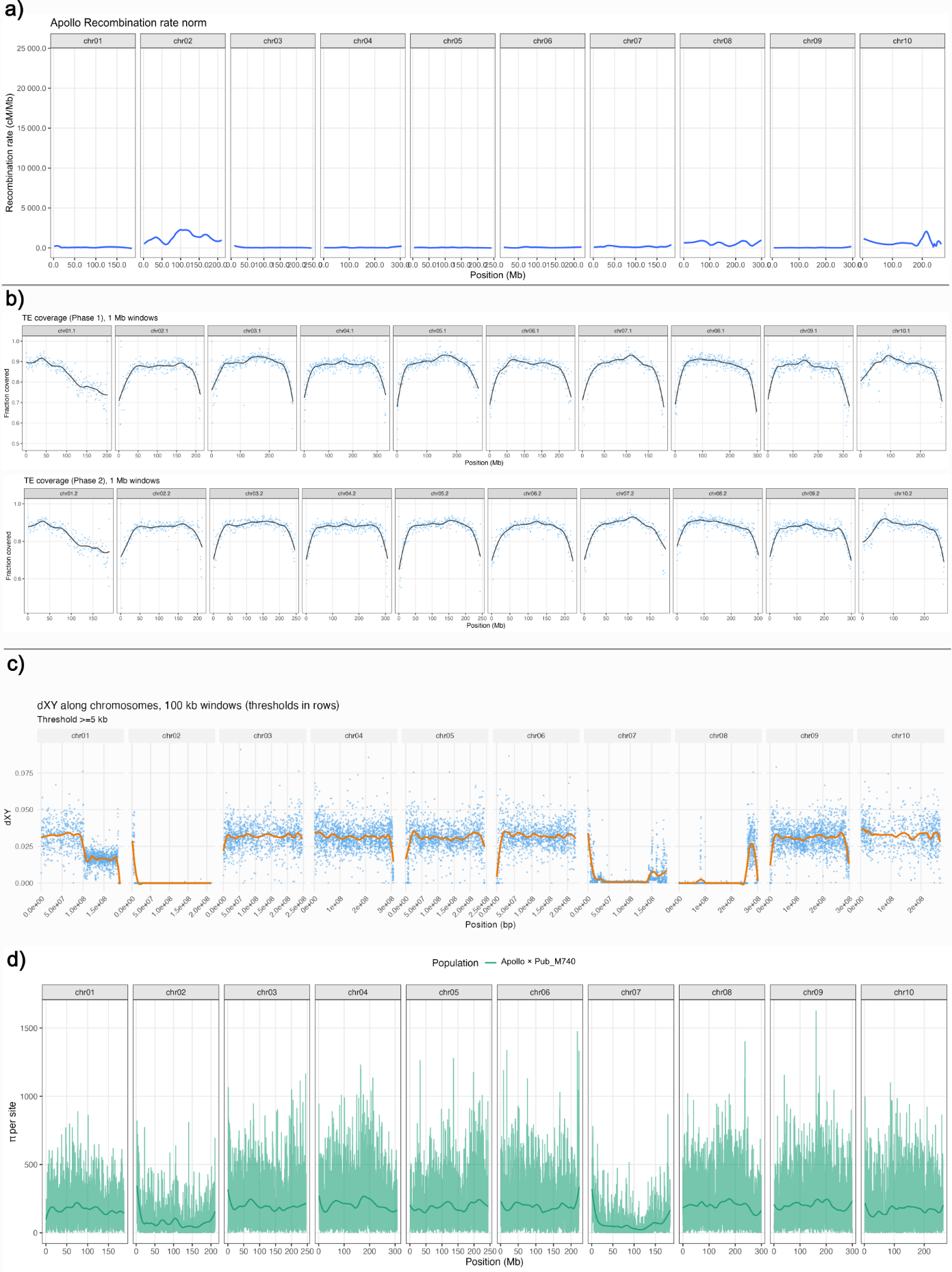


**Supplementary Fig. 13. Genome-wide recombination, diversity, and divergence patterns in cv. Apollo.** a) Recombination rate (Apollo maternal map, *n* = 184). Recombination is nearly absent across all chromosomes, with only minor peaks on chr02 and chr10. These peaks reflect, respectively, locally uniform diversity on chr02 and reduced marker density on chr10. Overall, recombination is strongly suppressed genome wide. b) Genome-wide Transposable Elements (TE) coverage across cv. Apollo haplotypes. TE density in 1 Mb windows shows an inverted U-shaped distribution, with TE-rich pericentromeres and low TE levels toward chromosome tips, consistent with the high gene-dense distal regions of the chromosomes. c) Haplotype divergence (dXY) between Apollo phase 1 and phase 2 assemblies (100 kb windows). dXY is elevated and relatively uniform across most chromosome arms but drops sharply within pericentromeric regions. These troughs approach zero, indicating long-term sequence conservation and reduced haplotype divergence in centromeric domains. Together, these profiles reflect strong structural and evolutionary constraints across the Apollo genome. d) Nucleotide diversity (π) in the Apollo × PubM_740 F₁ (*n* = 180). π is markedly reduced across broad central regions of all chromosomes and increases toward distal ends, a pattern consistent with large pericentromeric domains. Distal arms show higher diversity, whereas chromosomes with shared ancestry (e.g. chr02 and chr07) display uniformly low π. Source data are provided as a Source Data file.


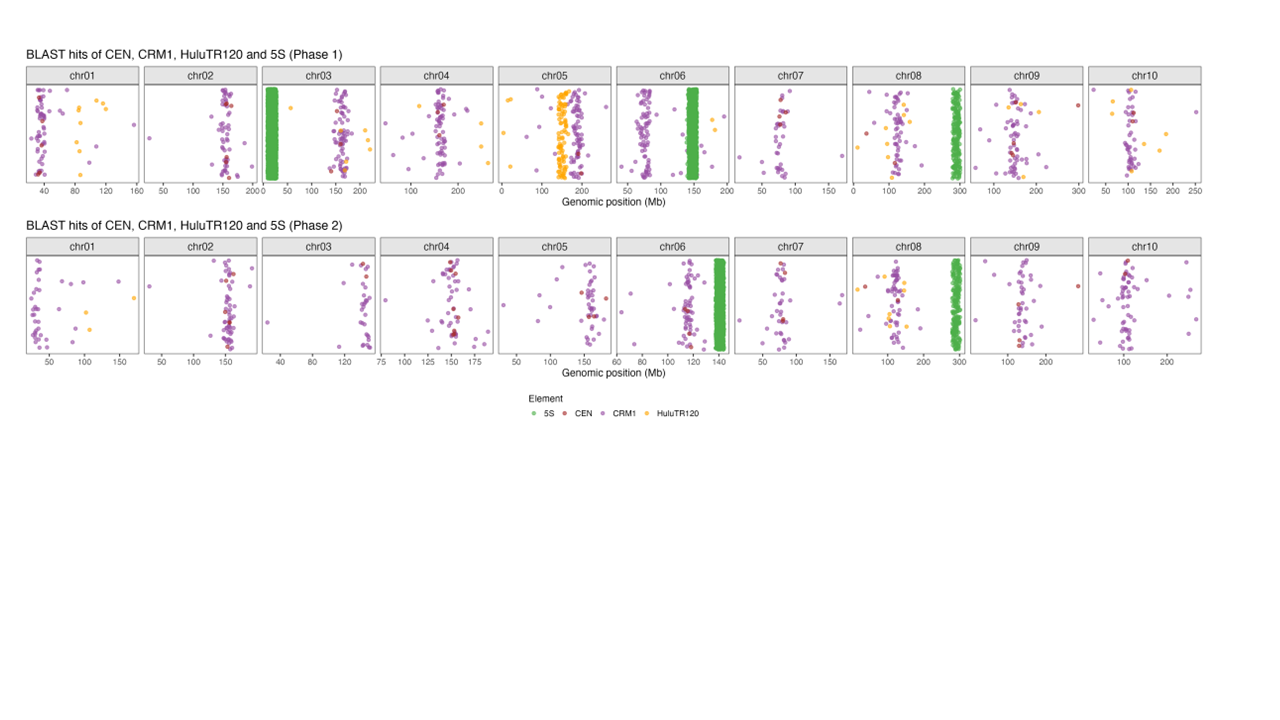


**Supplementary Fig. 14. Distribution of CEN, CRM1, HuluTR120, and 5S rDNA across Apollo haplotypes.** Chromosomal distribution of four key repeat families, CEN (SaazCEN), CRM1 (SaazCRM1), HuluTR120 (MN537570), and 5S rDNA (MN537579), based on consensus satellite sequences from https://tandem.bu.edu and BLAST alignments to the Phase 1 and Phase 2 Apollo pseudomolecules. BLAST searches were performed using an e-value threshold of 1e−20 and minimum sequence identity of 50%. HuluTR120 hits were further filtered to retain alignments with ≥ 97% identity and full query coverage. 5S rDNA loci were identified from BLAST hits filtered for query coverage > 95% and alignment length ≥ 5 kb. Hop centromeres are TE rich and highly dynamic, reflecting rapid evolution and strong structural heterogeneity among chromosomes. This structural complexity is consistent with meiotic irregularities during metaphase that may lead to unbalanced gametes. The distribution patterns shown here align closely with cytogenetic observations reported previously^16^. In Saaz, 5S rDNA localizes to chromosomes 2 and 5. In the Apollo genome, these signals map to chr08.2 and chr06.2 in Phase 2, and to chr08.1, chr06.1, and chr03.1 in Phase 1. Saaz chromosome 2 represents a notable exception within the chromosome complement, since its centromere is dominated by the satellite Saaz293 rather than SaazCEN or SaazCRM, and its pericentromeric domain is enriched for HuluTR120. This unusual centromere composition contributes to meiotic instability and the characteristic non-Mendelian segregation patterns observed for chromosome 2. Source data are provided as a Source Data file.


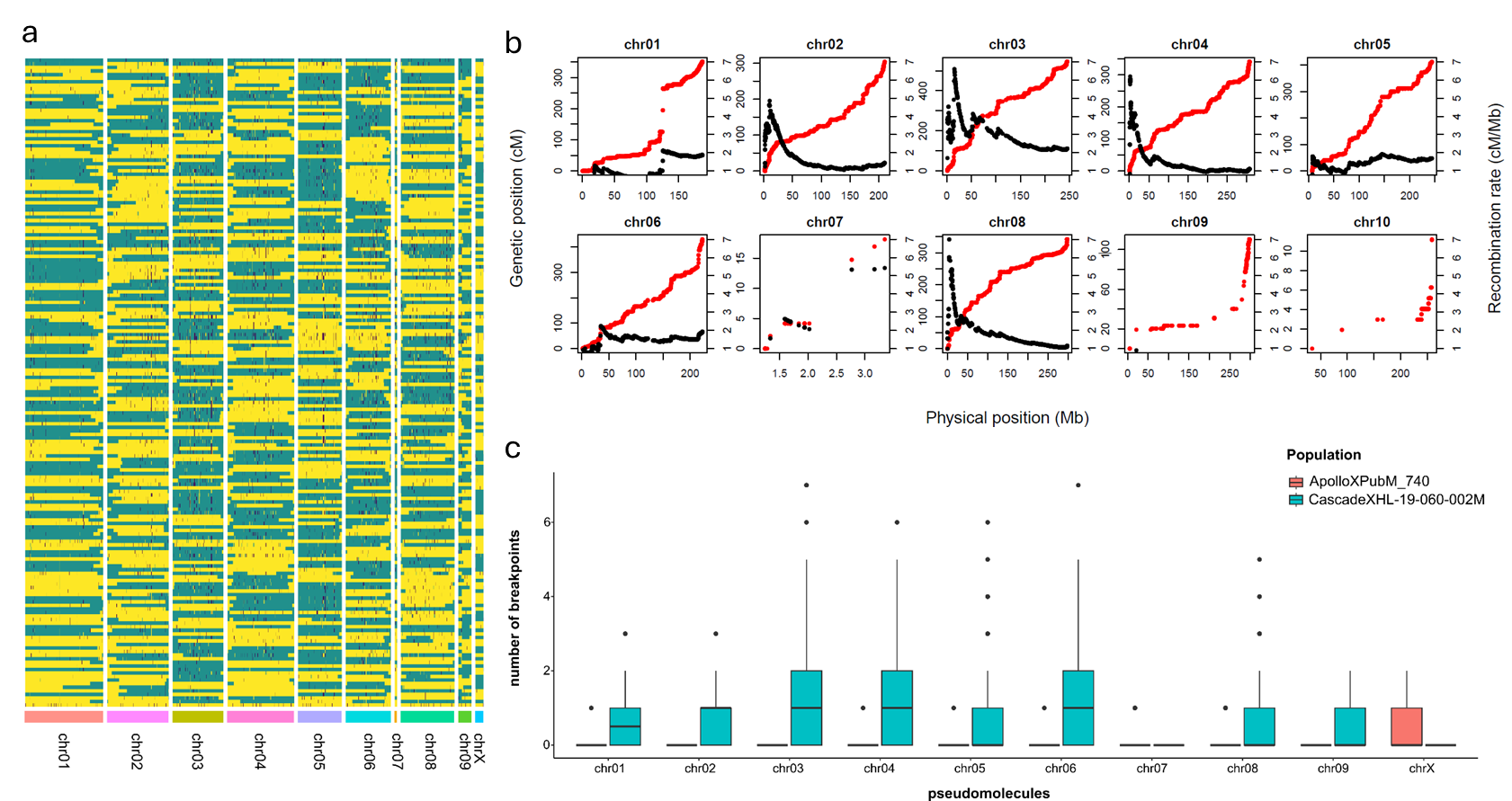


**Supplementary Fig. 15. Biparental mapping in the Cascade × HL-19-060-002 population.** a) Graphical genotypes of 1:1 segregating markers that are heterozygous in Cascade and homozygous in HL-19-060-002 in the Cascade × HL-19-060-002 population (*n* = 182). b) Plots of genetic position vs. physical position (red dots) and recombination rate vs. physical position (black dots) for markers heterozygous in Cascade and homozygous in HL-19-060-002M in the Cascade × HL-19-060-002M population (*n* = 182). c) Number of breakpoints per chromosome in the Apollo × PubM_740 and the Cascade × HL-19-060-002 populations. Data are shown for each pseudomolecule as box-and-whisker plots with all individual observations overlaid, and colors indicate the population. In each boxplot, the centre line indicates the median, the lower and upper bounds of the box indicate the 25^th^ and 75^th^ percentiles, and the whiskers extend to the smallest and largest values within 1.5 times the interquartile range from the box. Points beyond the whiskers are shown as outliers. Sample sizes: are *n* = 186 for the Apollo x PubM_740 and *n* = 166 for the Cascade × HL-19-060-002M population Source data are provided as a Source Data file.

Supplementary Fig. 16. A heatmap showing proportion of different chromosome pairs observed in hop cultivars. 243 accessions, of which 8.64%, 12.35%, 16.46%, 29.22%, and 33.33% were assigned as breeding lines, *H. lupuloides, H. neomexicanus*, commercial cultivars, and *H. lupulus*, respectively, were GBS genotyped. Chromosomes pairs from phase 2 were found in higher proportion compared to respective pair from phase 1. This maybe because of frequent use of *Humulus lupulus* males in breeding programs. Lowest proportion of chr09.1 and chr06.2 combination was observed in current panel indicating negative selection. Source data are provided as a Source Data file.


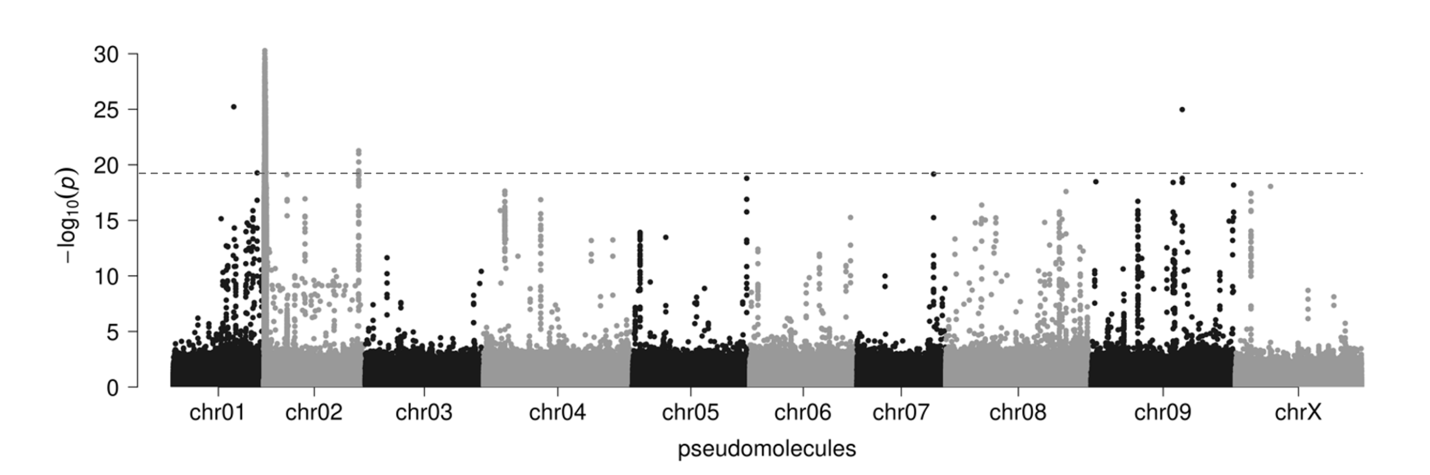


Supplementary Fig. 17. GWAS for DMR in the Apollo × PubM_740 population. The Manhattan plot shows association results across hop pseudomolecules. The y-axis shows −log10(Wald test P values) and the dashed horizontal line indicates the Bonferroni significance threshold. The analysis identified a 1.8 Mb region on chr02 between bps 3,101,412 and 4,994,557 associated with DMR in hop. We used linear mixed models with SNP filters of ≤ 10% missing data, MAF > 0.01, an IBS-based kinship matrix, two-sided tests, and a Bonferroni significance threshold of α = 0.05. *n* = 186. Source data are provided as a Source Data file.

**
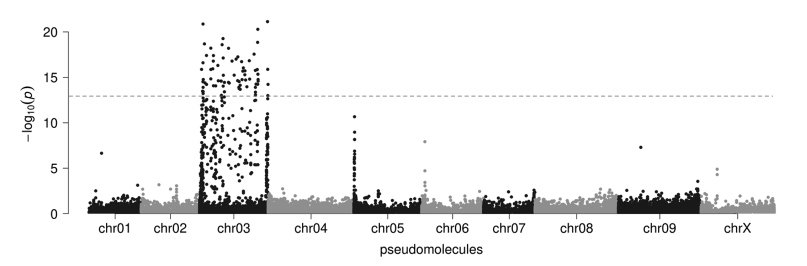
**

Supplementary Fig. 18. GWAS for PMR in the Zenith × USDA21058M population. The Manhattan plot shows association results across hop pseudomolecules. The y-axis shows −log_10_(*P*) (Wald test), and the dashed horizontal line indicates the Bonferroni significance threshold. We used linear mixed models with SNP filters of ≤ 10% missing data, MAF ≥ 0.01, an IBS-based kinship matrix, two-sided tests, and a Bonferroni significance threshold of α = 0.05. The analysis identified the entire pseudomolecule chr03 and a 1.5 Mb region on chr05 between bps 2,659,811 and 4,218,174 associated with PMR (*n* = 128). Source data are provided as a Source Data file.

Supplementary Fig. 19. Identification of pseudo-autosomal region on the sex chromosome (chrX) in hop. Chromosome-wise Fst analysis between 95 female and 20 male accession (*n* = 105) was carried out and plotted against the physical position. A ~31Mb region on the chrX spanning positions 228,120,000 to 259,120,000 was identified as pseudo-autosomal region. Source data are provided as a Source Data file.

**
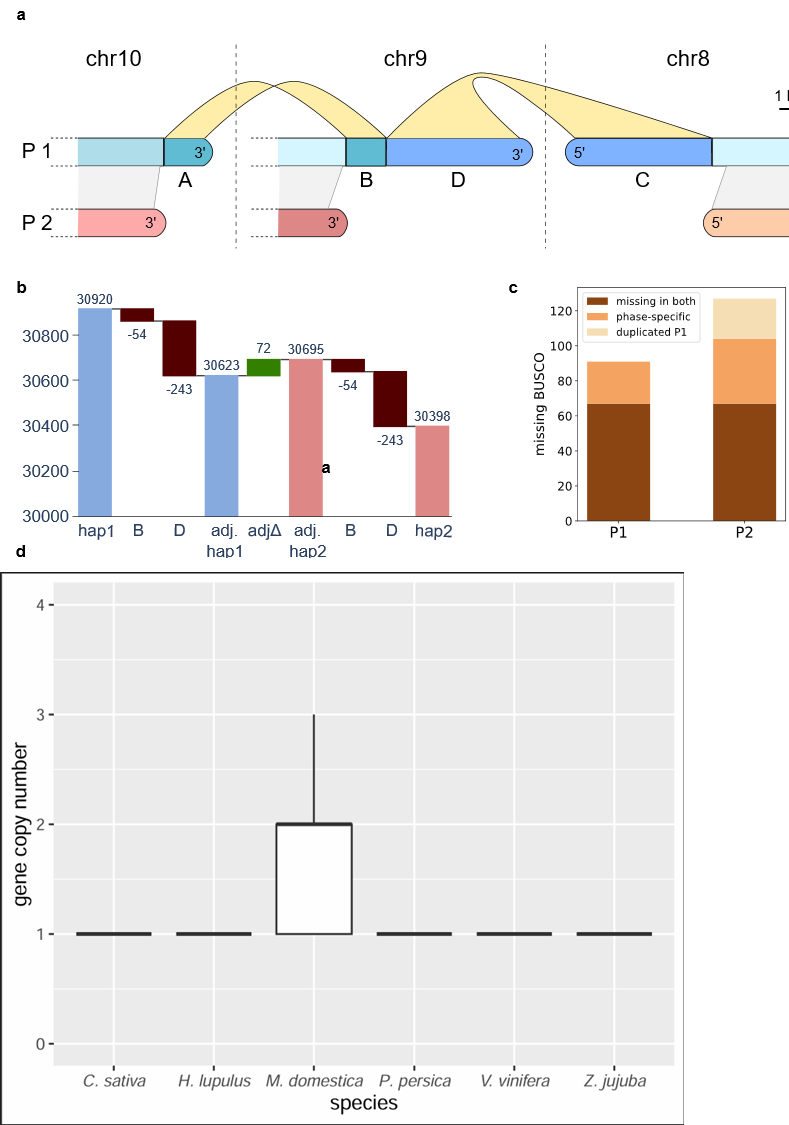
**

**Supplementary Fig. 20. Gene annotation** a) Intraphase Translocations. Synteny analysis identified two duplications on chromosome 9 of phase 1 (P1) which likely represent two small translocation events derived from chromosome 10 (A, B block pair) and chromosome 8 (C, D) of phase 2 (P2). b) The intra-phase translocation largely explains the difference (522 genes) in total gene annotations between phase 1 (30,920 genes) and phase 2 (30,398 genes): *in silico* transfer of block B and D genes (297 genes) from phase 1 to phase 2 reveals highly similar gene content between both phases with a total adjusted difference of 72 genes (adj∆). Y-axis is magnified and starts at 30,000 genes to illustrate stepwise subtraction (left half) or addition (right half) of phase 1 and 2, respectively. c) Blocks A-D of phase 1 (P1) contain 53 duplicated Busco genes of which 23 can complement missing Busco genes of phase 2 (P2) widely leveling out the observed difference of missing Busco genes between P1 (91 genes) and P2 (127 genes). Upon mapping gene models from phase 2 to phase 1, and vice versa, it was noted that approximately 10% of gene models from each phase remained unannotated. d) Absence of whole genome duplication. No significant evidence of whole genome duplications (WGD) affecting genome size across most Rosales lineages, including hops. Boxplots compare the distribution of gene family sizes across 11,220 orthologous groups with at least one gene copy in all species. In each boxplot, the centre line indicates the median, the lower and upper bounds of the box indicate the 25^th^ and 75^th^ percentiles, and the whiskers extend to the smallest and largest values within 1.5 times the interquartile range from the box. Data are displayed within the main range of 0–4 gene copies to visualize the predominant genome-wide copy number pattern. All species except *Malus domestica* show a median gene copy number of 1 and an interquartile range of 0, whereas *M. domestica* shows an upward shift in gene copy number distribution, consistent with a previously reported whole genome duplication^17^. Source data are provided as a Source Data file.


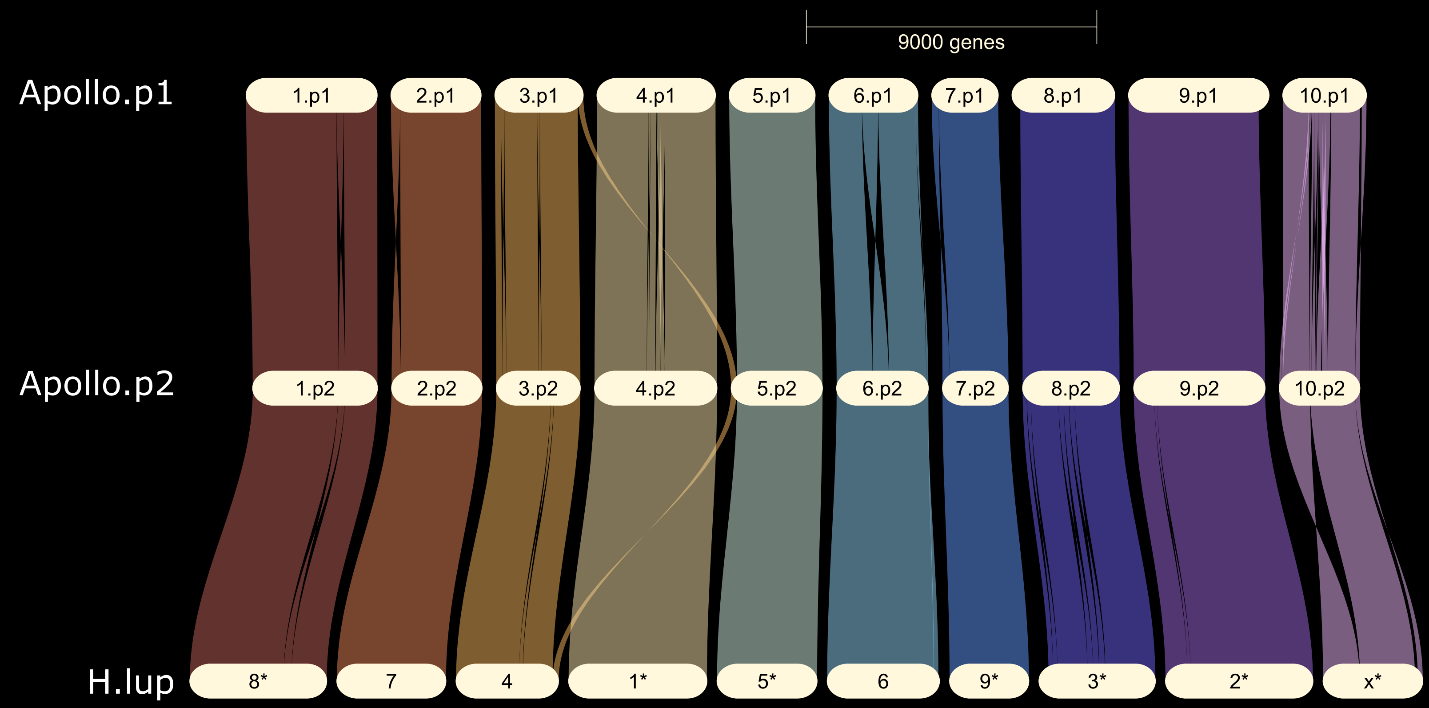


Supplementary Fig. 21. Comparison of the phased cv. Apollo genome assembly and the haploid assembly drHumLupu1.1 of European hop (NCBI GCF_96316925.1). Orthologous relationships were computed using GENESPACE*^18^* to visualization of the synteny (conserved gene order) identified between both assemblies (HOPhap1: Apollo phase 1, HOPhap2: Apollo phase 2, H. lup: drHumLupu1.1 haploid assembly). * indicate chromosomes inverted from their published orientation. Source data are provided as a Source Data file.


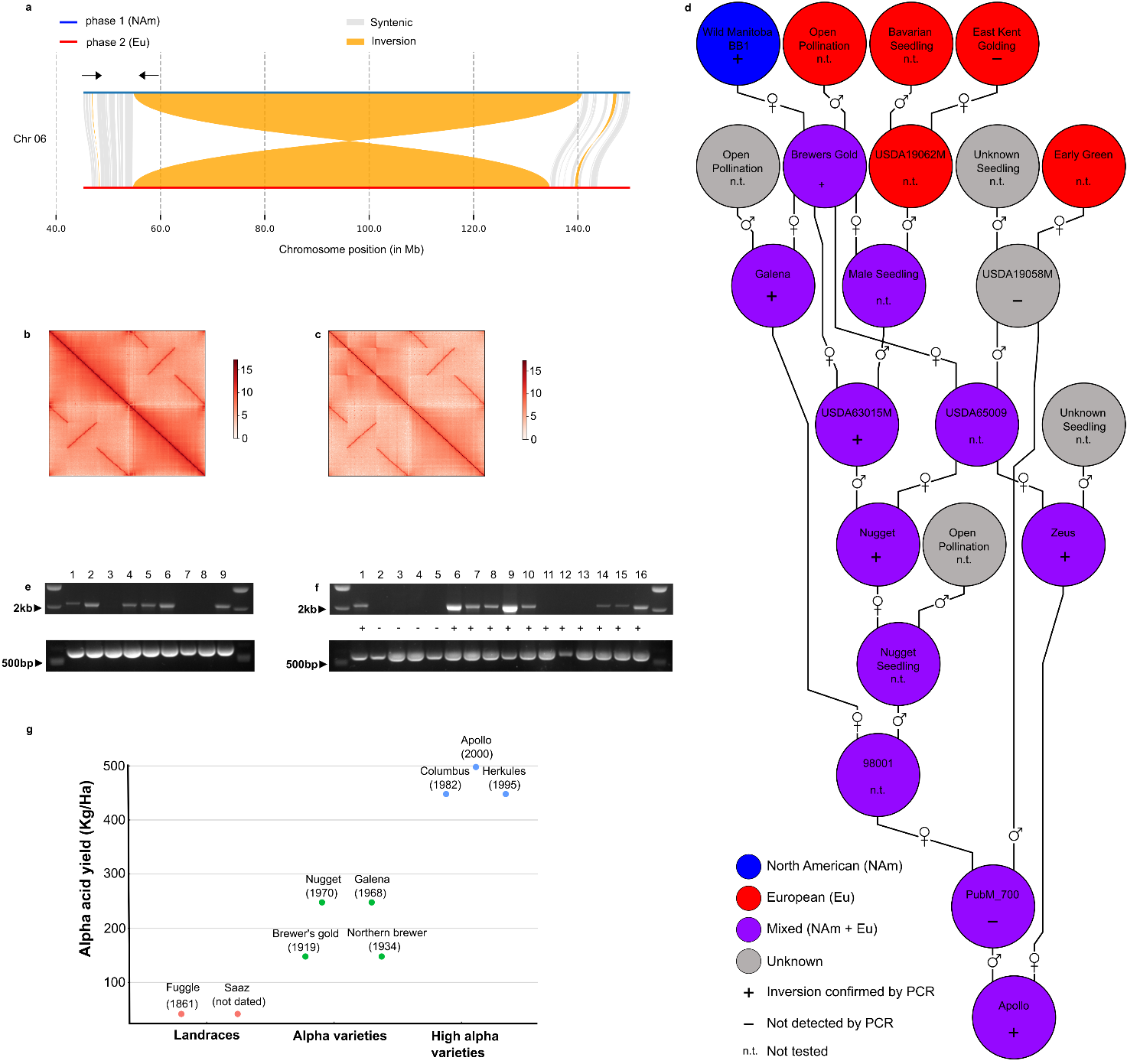


Supplementary Fig. 22. Inversion present on the NAm phase of chr06. a) Schematic overview on the inverted region and the PCR strategy to trace the inversion present on the NAm phase of chr06. The region between positions 54872166 and 140780292 on chr06.1 (85.9081 Mb) and positions 54674504 and 134556775 on chr06.2 (79.8823) was found to be inverted. Arrows schematically show binding sites for primers specifically amplifying the inversion breakpoint on chr06.1 of Apollo. b) Hi-C contact matrix generated by aligning Hi-C reads from Apollo against the phased Apollo assembly. Discontinuity in the line parallel to the diagonal indicates the inverted region. c) Hi-C contact matrix generated by aligning Hi-C reads from Cascade against the phased Apollo assembly. The discontinuity on the diagonal line in phase1 of chr06 indicates that the inversion is present on phase1 of chr06 in the Apollo assembly. d) Tracing a 85 Mb inversion on chr06 phase1 in the known pedigree of *Humulus* cv. Apollo*^19,20,21,22^* by PCR. The most distant ancestor of Apollo, in which the inversion could be detected is cv. Brewer’s Gold. n.t. = not tested, + = inversion detected by PCR, − = inversion not detected by PCR, blue: North American hop genotype, red: European hop genotype, purple: hop genotype with mixed North American and European pedigree, grey: hop genotype with unknown origin, OP = open pollination. e) PCR reactions to trace chr06.1 breakpoint by PCR in available genotypes present in the pedigree of Apollo (upper panel) and positive control (lower panel) (cultivars: Lane 1 = Apollo; Lane 2 = Zeus; Lane 3 = PubM_740; Lane 4 = Nugget; Lane 5 = M63015; Lane 6 = Galena; Lane 7 = M19058; Lane 8 = East Kent Golding; Lane 9 = Brewer’s Gold). Each sample was analysed by PCR a minimum of three times, yielding same result each time. f) Tracing of the chr06.1 breakpoint by PCR in commercial *Humulus* cultivars with Brewer’s Gold in their pedigree (upper panel) positive control (lower panel) (“+” = Brewer’s Gold is present in pedigree; “−“ = Brewer’s Gold not present in pedigree; cultivars: Lane 1 = Brewer’s Gold; Lane 2 = Bullion; Lane 3 = Cascade; Lane 4 = Comet; Lane 5 = Saaz; Lane 6 = Eroica; Lane 7 = Galena; Lane 8 = Horizon; Lane 9 = Triumph; Lane 10 = Centennial; Lane 11 = Chinook; Lane 12 = Olympic; Lane 13 = Crystal; Lane 14 = Glacier; Lane 15 = Tahoma; Lane 16 = Citra) Each sample was analysed by PCR a minimum of three times, yielding same result each time. g) α-acid content in several commercial high-α *Humulus* cultivars released since 1919 depicted as kg α-acid harvested per ha. The cumulative α-acid content data were calculated based on the long-term α quantities produced (kg α acid/ha) from the annual average yields (kg raw hops/ha) and the annual average α contents (%) in the years 2008 to 2023 ([www.hopslist/hops.com](http://www.hopslist/hops.com)). The α yields (kg ha^-1^) were calculated from the average α values (%) and the average raw hop yields (kg ha^-1^). Prior to the release of Brewer’s Gold, only landraces were cultivated in Europe, which had a low α-acid yield. All cultivars shown which were released after 1919 have Brewer’s Gold in their pedigree*^20^*. Source data are provided as a Source Data file.

Supplementary Fig. 23. Gene family extension/contraction analysis reveals gene family expansions specific to the Cannabaceae. Orthologous gene family expansions and contractions during Rosales evolution. Shown are significant (*P <* 0.05) expansions (blue) or contractions (red) of orthologous gene families with gene copies in all species, in addition to expansions (green) and contractions (orange), of orthologous gene families with at least one copy in each Cannabaceae species but missing a copy in one or more of the other species.


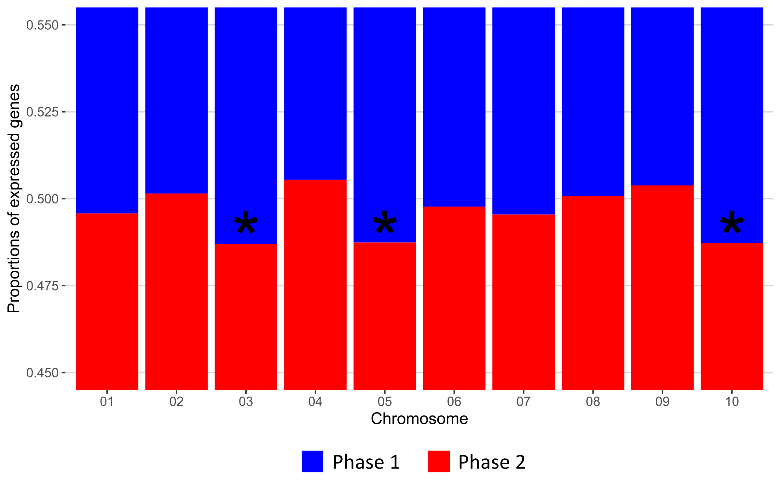


Supplementary Fig. 24. Genome-wide allelic expression analysis. To achieve a comprehensive overview on allele-specific expression (ASE) for annotated genes in the Apollo genome, homo- and heterozygous gene pairs were identified based on coding sequence comparison and filtered by gene expression. For these 19725 ortholog gene pairs, expression data was used to conduct a test for significant differential expression between both phases during cone development. Gene pairs that showed significant ASE (adjusted *P <* 0.05) were solely accounted for their dominant gene, while all remaining pairs were marked having equal expression. For each chromosome and phase, the ratio of the total amount of ASE to the total number of present genes was calculated and subsequently summarized for all five developmental stages within a stacked bar plot to visualize the proportion of expressed genes in the context of ASE for every chromosome. Hereby, chromosomes 3, 5 and 10 show a distinct tendency in ASE towards phase 1, which was further confirmed to be significant with *P* = 0.001, 2 × 10^−4^ and 6 × 10^−4^, respectively (asterisks indicates *P <* 0.05) by applying a two-proportions Z-test for each chromosome. Source data are provided as a Source Data file.


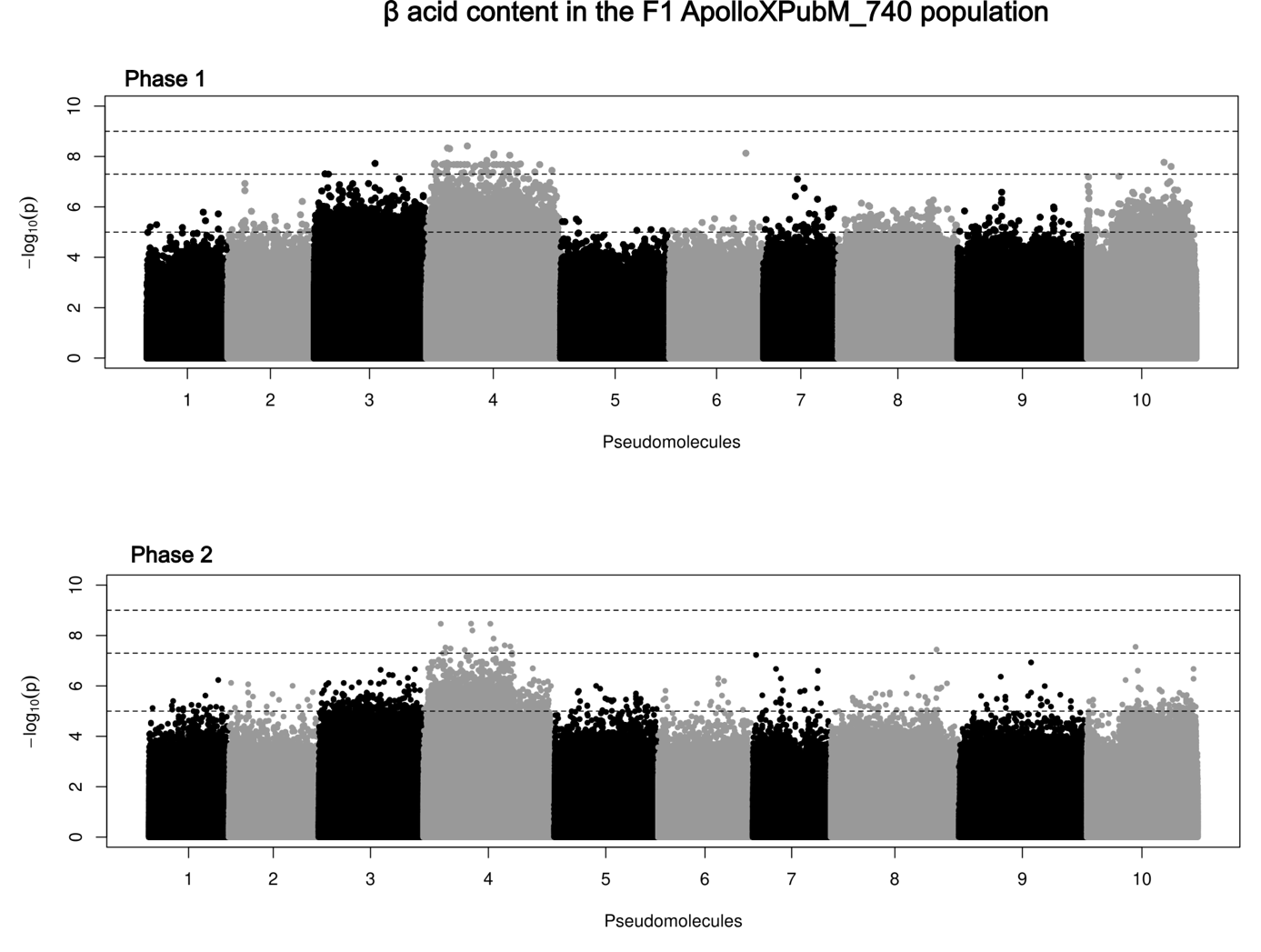


**Supplementary Fig. 25. GWAS for β acid BLUPs in the F1 Apollo × PubM_740 population.** Manhattan plots show association results for β acid content using the Phase 1 (top) and Phase 2 (bottom) Apollo reference haplotypes. In Phase 1, the pattern indicates that several genomic regions contribute to β acid variation, with clear signals on chr03, chr04, and chr10. In Phase 2, an association is observed on chr04. These results are complementary and suggest a polygenic architecture for β acid content. Transcription factors previously reported to be involved in the regulation of bitter acid biosynthesis^23^, including HlMYB3, HlMYB7, HlMYB8, and the putative HlMYB78, are located on chr04.1 and chr04.2 and co-localize with the association signals. In both analyses we used the linear mixed models with SNP filters of ≤ 10% missing data, MAF ≥ 0.01, an IBS-based kinship matrix and two-sided tests. Dotted horizontal lines indicate nominal significance thresholds: *P* < 1 × 10⁻⁵ (−log_10_ (*P*) > 5), *P* < 1 × 10⁻⁸ (−log_10_ (*P*) > 7.3), and *P* < 1 × 10^−^⁹ (−log_10_(*P*) > 9). Source data are provided as a Source Data file.

**
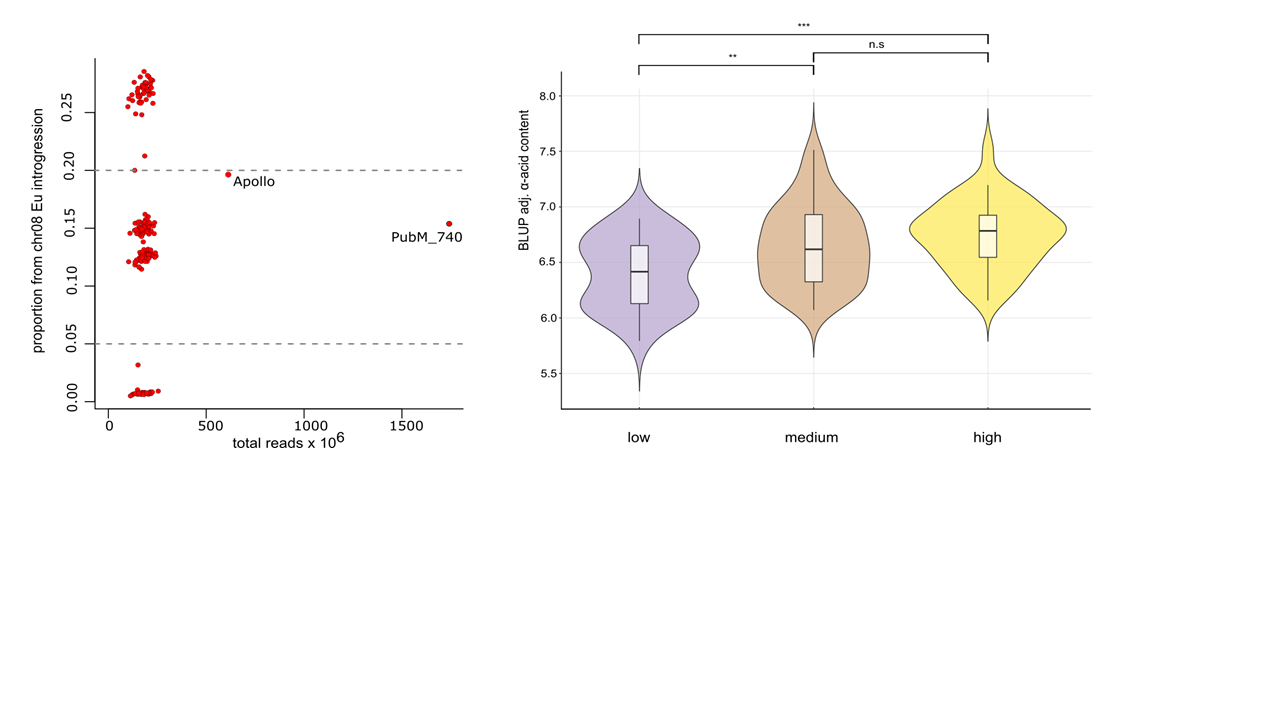
**

**Supplementary Fig. 26. Eu introgression on chr08 and its impact on α-acid level in the ApolloXPub M740 population** **(*n* =128).** a) Detection of the introgressed Eu region on chr08 through diagnostic k-mers. The analysis identified three distinct clusters corresponding to individuals carrying none, one or two copies of the introgression and showing 1:2:1 segregation. The relative frequency of k-mers (low, medium, and high) from the introgressed region on chr08 in the Apollo × Pub_M_740 population is separated by dotted lines. b) Total α-acid BLUP values for individuals in each chr08 introgression class, low, medium, and high. The violin shape represents the data distribution within each group. In each boxplot, the centre line indicates the median, the lower and upper bounds of the box indicate the 25^th^ and 75^th^ percentiles, and the whiskers extend to the smallest and largest BLUP-adjusted α-acid values within 1.5 times the interquartile range from the box. Group sizes were low (n = 26), medium (n = 63), and high (n = 39). Significance between groups was assessed using one-way ANOVA followed by Tukey’s HSD multiple-comparison test; brackets indicate pairwise comparisons, with **P < 0.01, ***P < 0.001, and n.s. indicating not significant. Source data are provided as a Source Data file.


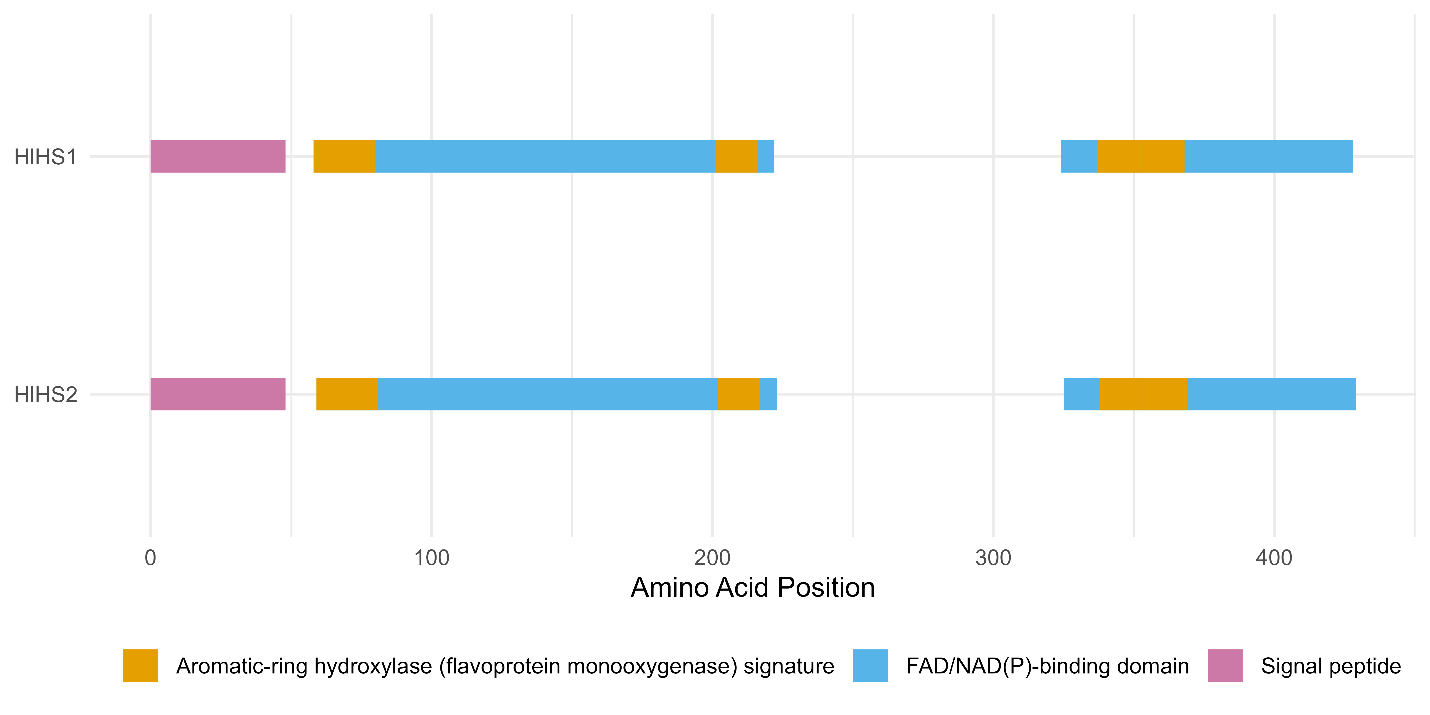


**Supplementary Fig. 27 HlHS1 and HlHS2.** Domain architecture of HlHS1(KJ398144.1) and HlHS2 (KJ398145.1). Both proteins are predicted to be plastid-targeted based on signal peptide analysis using DeepLoc2.1*^24^*, with localization likelihoods of 0.9802 for HlHS1 and 0.9753 for HlHS2. Additionally, DeepLoc2.0 predicts both proteins as potential peripheral membrane proteins (likelihood 0.67). InterPro domain analysis*^25^* identifies two FAD/NAD(P)-binding domains and three flavoprotein monooxygenase signature sites in the sequences of both enzymes. The domain annotations identified by InterPro are consistent with sequence homology results obtained via NCBI BLASTp*^26^*. The closest homologues in the non-redundant protein database include uncharacterized, predicted plant flavoprotein monooxygenases. Although these sequence-based predictions are consistent with a potential role for HlHS1 and HlHS2 as oxidases in alpha-acid biosynthesis, the heterologous production of alpha-acids such as humulone has, despite significant efforts, remained largely unsuccessful. Prior work in S*accharomyces cerevisiae* demonstrates that while trace amounts of the precursor lupulone can be produced, conversion to humulone is inefficient and non-quantitative*^27,28^*. Several factors likely contribute to this limitation. First, lupulone is highly hydrophobic and may not be stably synthesized or stored in the intracellular environment of heterologous hosts, unlike in *H. lupulus*, where it is secreted directly into glandular trichomes. Second, lupulone possesses antimicrobial properties that may compromise cell viability by disrupting organelle membranes. Third, the enzymes involved in the downstream conversion to humulone may require a hydrophobic microenvironment for proper activity. Finally, these enzymes might also rely on continuous product removal to remain functional; in the absence of effective secretion, product accumulation could lead to feedback inhibition or enzyme inactivation. Together, these factors present substantial physiological and biochemical barriers to alpha-acid biosynthesis outside of hops.

Supplementary Fig. 28. MALDI-MSI analysis of key metabolites in cv. Apollo. a) fluorescence image of cross section of a hop cone at 2 weeks after flowering (WAF), before matrix application. b) and c) MS images of ions with *m/z* values corresponding to [M-H]- of the indicated compounds, b) general metabolites, c) bitter acids and prenylated phenylpropanoids. As 6- and 8-prenylnaringenin are structural isomers with identical sum formula, these cannot be differentiated in this MALDI-MSI analysis. The remaining compound annotations assume that there are no structural isomers present in the cones. d) Overlays of the MS images of *m/z* values from c) with *m/z* value corresponding to glutamic acid. All images are shown without cut-off in the lower or higher intensities, with color bar for illustration of maximum and minimum intensity values. For overlapping images, glutamic acid was used to show the structural shape of the sample (strig, bracts and bracteoles). MS images were plotted using *m/z* values normalized to total ion current (TIC) and with a tolerance of ± 10 ppm to the assigned *m/z* value. Scale bar corresponds to 3 mm. Experiment was done in respectively 2 and 3 replicates from two different growth experiments, and the data shown are a representative example.


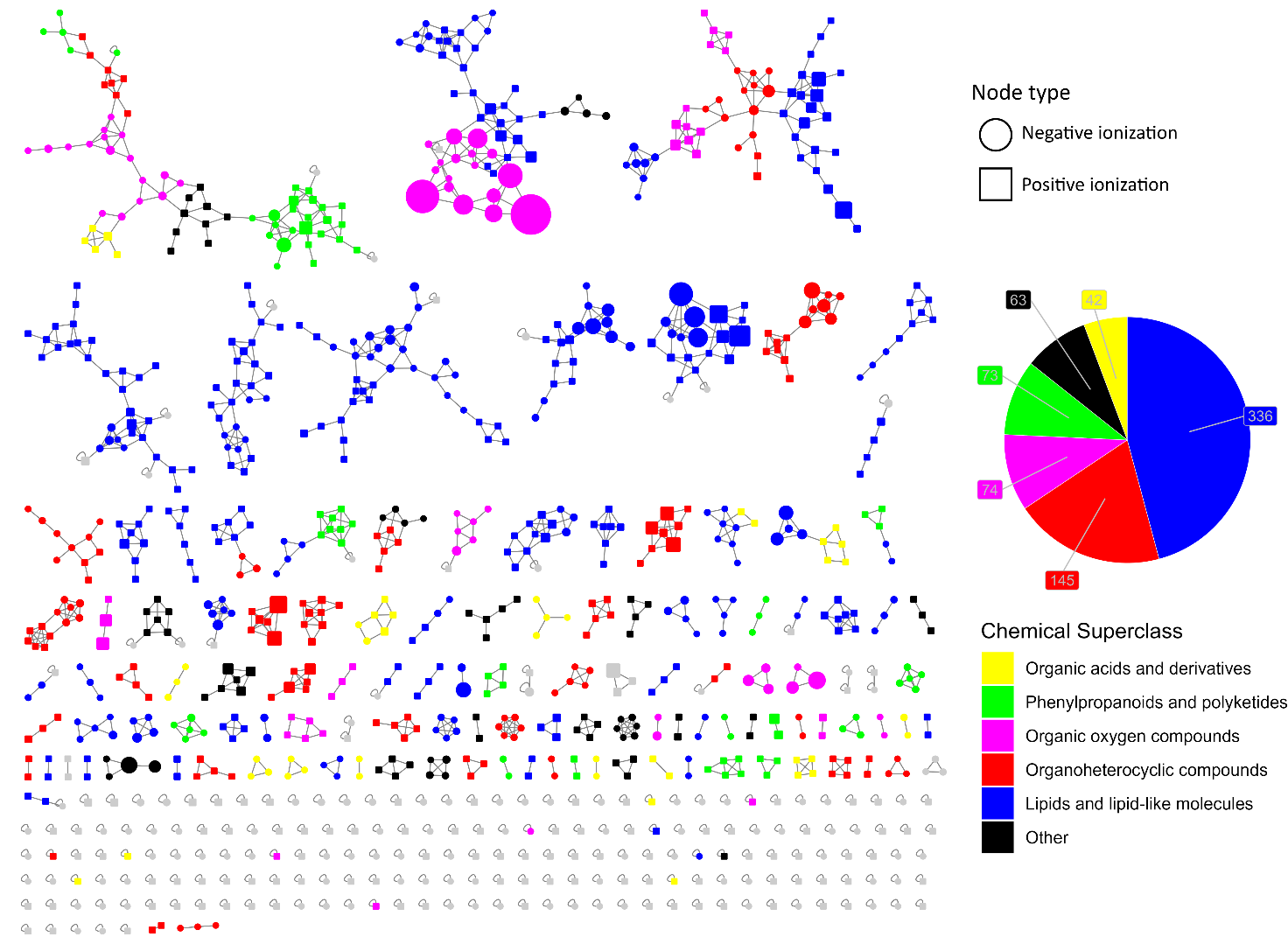


Supplementary Fig. 29. Global molecular network of the non-volatile chemical space during hop cone development. The global molecular network representing the non-volatile chemical space of hop cone development comprises clusters of chemical features (subnetworks) that display structurally related putative metabolites, as well as single nodes (also called singletons), which represent features that did not show spectral similarity to others under the given set of parameters. Node size was relatively scaled to the sum of the respective signal intensities determined in all five developmental stages of the gland fraction after averaging the replicates. The generated network served as a foundation for chemical dereplication efforts involving public, in-house, and in silico spectral libraries and subsequent Network Annotation Propagation (NAP) that gave rise to a global chemical classification. The presented network displays NAP-generated chemical superclass prediction for each node with selected classes highlighted in given colors, whereas singletons are displayed in grey. This network was generated on the merge of positive and negative ionization networks data. Subnetworks with mixed chemical classes are therefore the product of involving spectral data from both ionization dimensions, which informs and supports the insight into chemical identity by adding more structural information. The pie chart on the right displays the total count of nodes for relevant chemical superclasses highlighted in this network, after classification score cutoff was applied (1^st^ quantile of chemical superclass score = 0.29). This indicates a dominance of lipid-related compounds in the hop cone chemical space, mostly due to the abundance of terpenes as well as highly prenylated metabolites, which are assigned to the chemical class of prenol lipids. Source data are provided as a Source Data file.


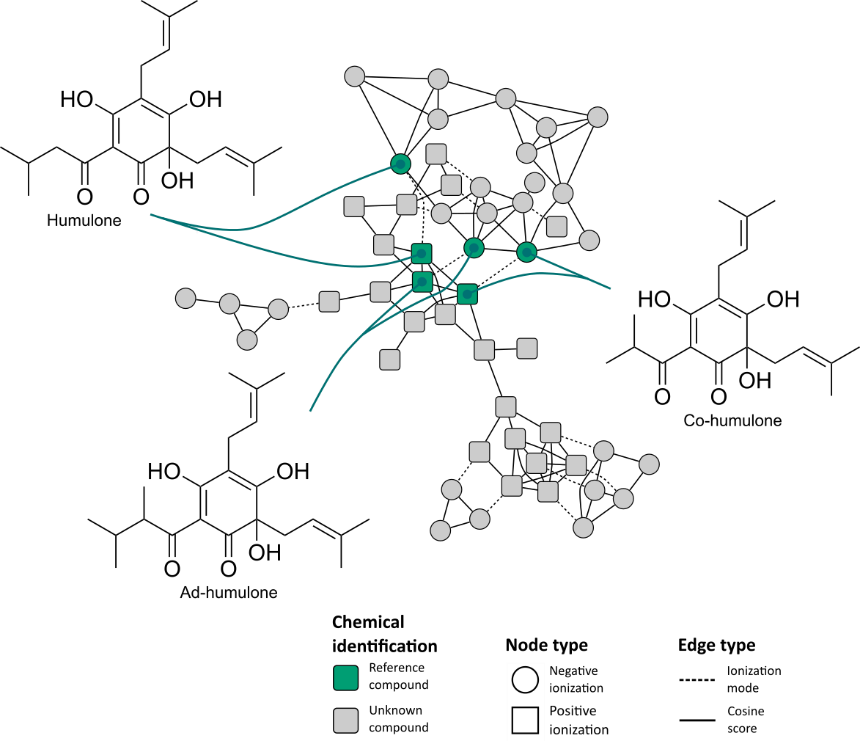


**Supplementary Fig. 30. Diversity of humulone chemistry and associated polyketide synthase genes.** The chemical family of humulone is composed of 53 analogous compounds and was isolated from a molecular network generated on non-volatile chemical data, extracted from glandular fractions sampled over five developmental stages in cv. Apollo. This network is composed of structurally related chemical features represented as nodes, which are connected via edges that display structural similarity. To extend the chemical insight of this approach, spectral data was generated in both ionization modes, positive and negative, as displayed by squared and circular shaped nodes, respectively. Three chemical features could be annotated by matching their spectral fingerprint to a reference compound spectrum (green node), highlighting the major humulone analogs: Humulone, Ad-humulone and Co-humulone. All remaining chemical features represent putative compounds that share a similar molecular structure to the identified humulones, while detailed structural information remains unknown (grey node). Source data are provided as a Source Data file.

**
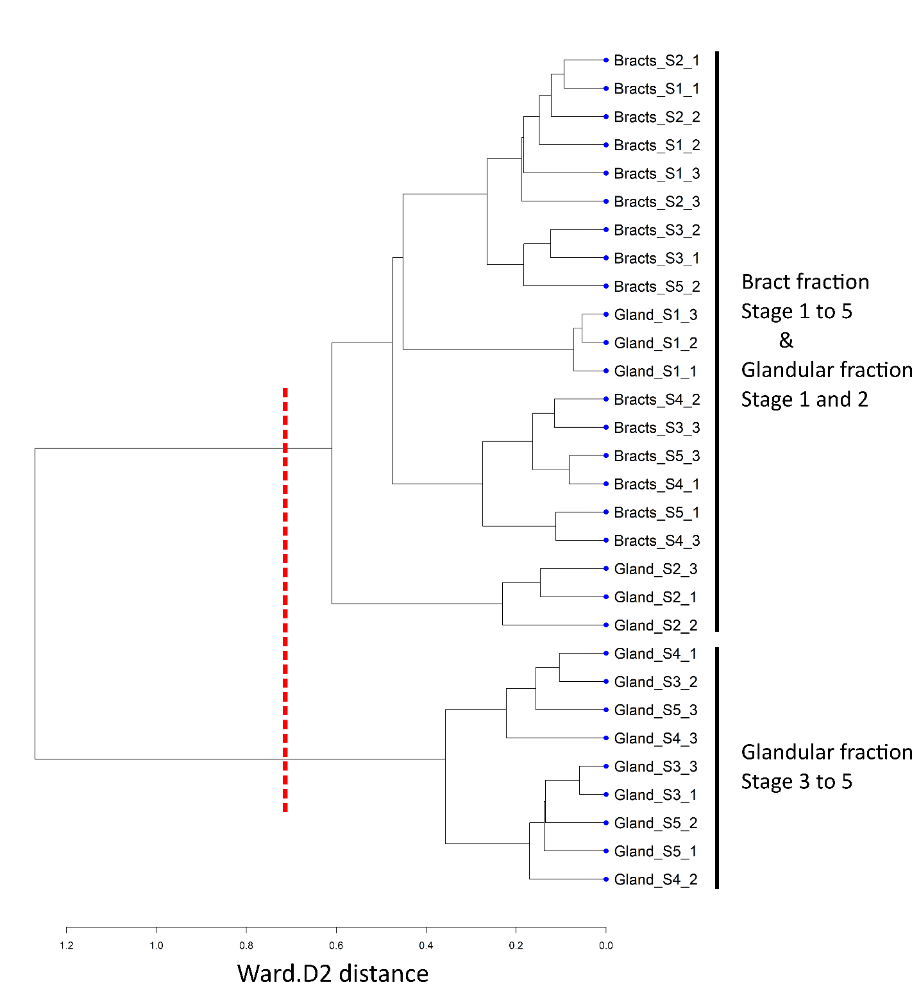
**

Supplementary Fig. 31. Hierarchical cluster analysis of volatile and non-volatile chemical features present during hop cone development. Gland and bract fraction sampled in triplicates throughout five stages (S1-S5) of hop cone development underwent chemical analysis to determine volatile (VC) and non-volatile (NVC) compounds. Hierarchical cluster analysis was conducted based on the merged set of combining 49 VC and 987 NVC, with the latter 424 and 563 NVC deriving from negative and positive ionization, respectively. The generated dendrogram displays chemical similarity among the sample set based on presence/absence information of each spectral feature that was clustered using the ‘Ward.D2’ agglomeration method. In this cluster analysis, samples from the gland fraction of stage 3 to 5 form a distinct subcluster, which suggests a specialized metabolite profile compared to the remaining samples. This chemical diversification in the glandular fraction appears to begin already in stage 2, while glands of stage 1 together with the less specialized total bract fraction form another subcluster. Source data are provided as a Source Data file.


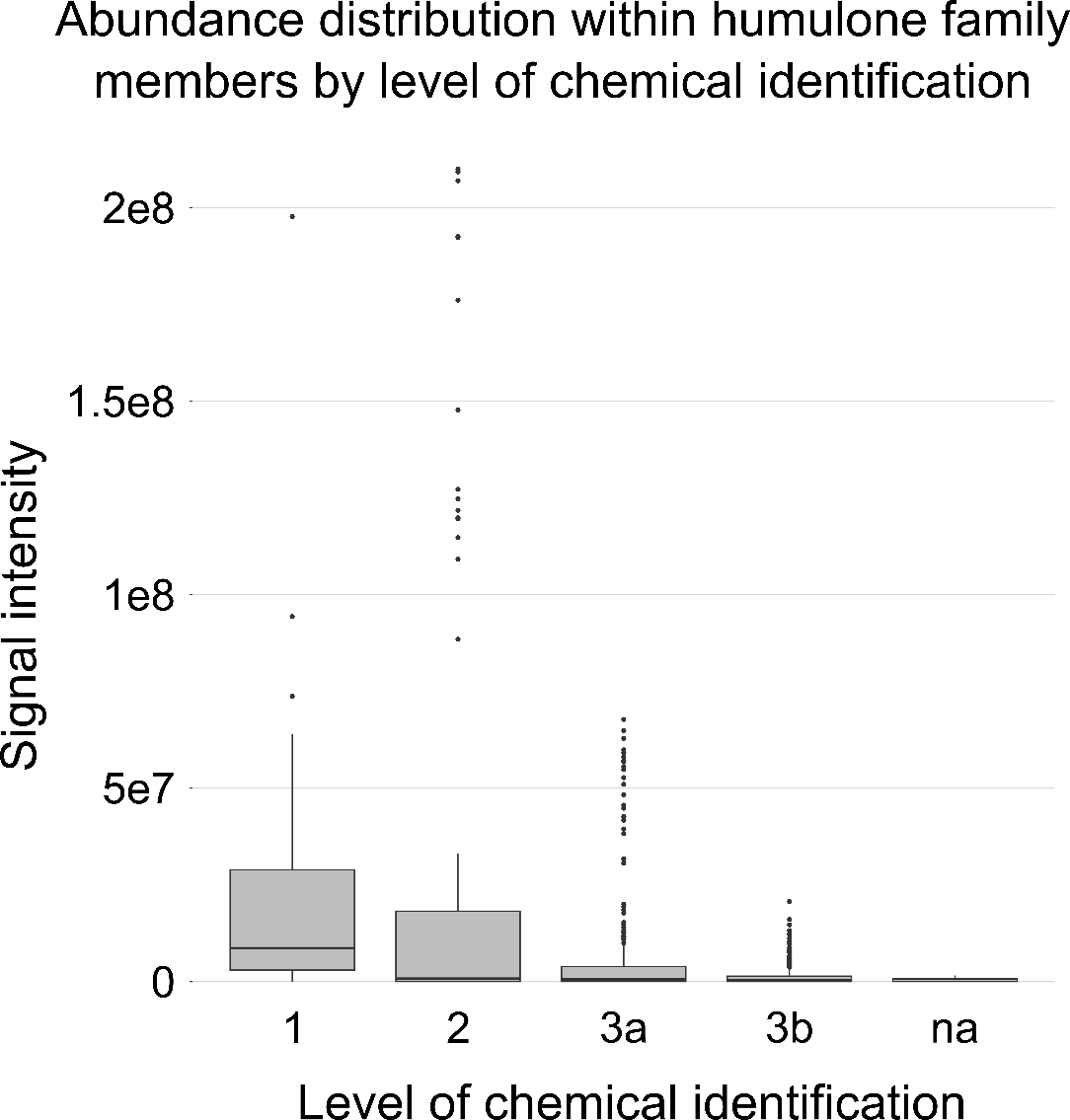


**Supplementary Fig. 32**. **Abundance distribution of humulone chemical family members across different levels of identification**. The chemical family around the described humulone analogs, N-, Ad- and Co-humulone, comprises 53 spectral features determined in positive and negative ionization, while 47 features could only be associated to the humulone scaffold with their true structure yet to be elucidated. To highlight their relevance within this chemical space, the signal intensity of all features was plotted according to their level of identification. Level 1 comprises the above-mentioned known humulone analogs (6 features), while undescribed analogs were annotated on different levels of identification, with level 2 (7 features), level 3a (14 features), level 3b (25 features) and no annotation (na) associated to a single feature in this chemical family. The latter group does contain features with elevated abundance, compared to the main analogs, and thus underlines its relevance during cone development in cv. Apollo. Boxplots were generated using the default settings of the ggplot2 package in R. The centre line indicates the median. The box limits represent the 25^th^ and 75^th^ percentiles (interquartile range, IQR). Whiskers extend to the smallest and largest observations within 1.5 × IQR of the lower and upper quartiles. Observations beyond the whiskers are plotted as individual outliers. Source data are provided as a Source Data file.


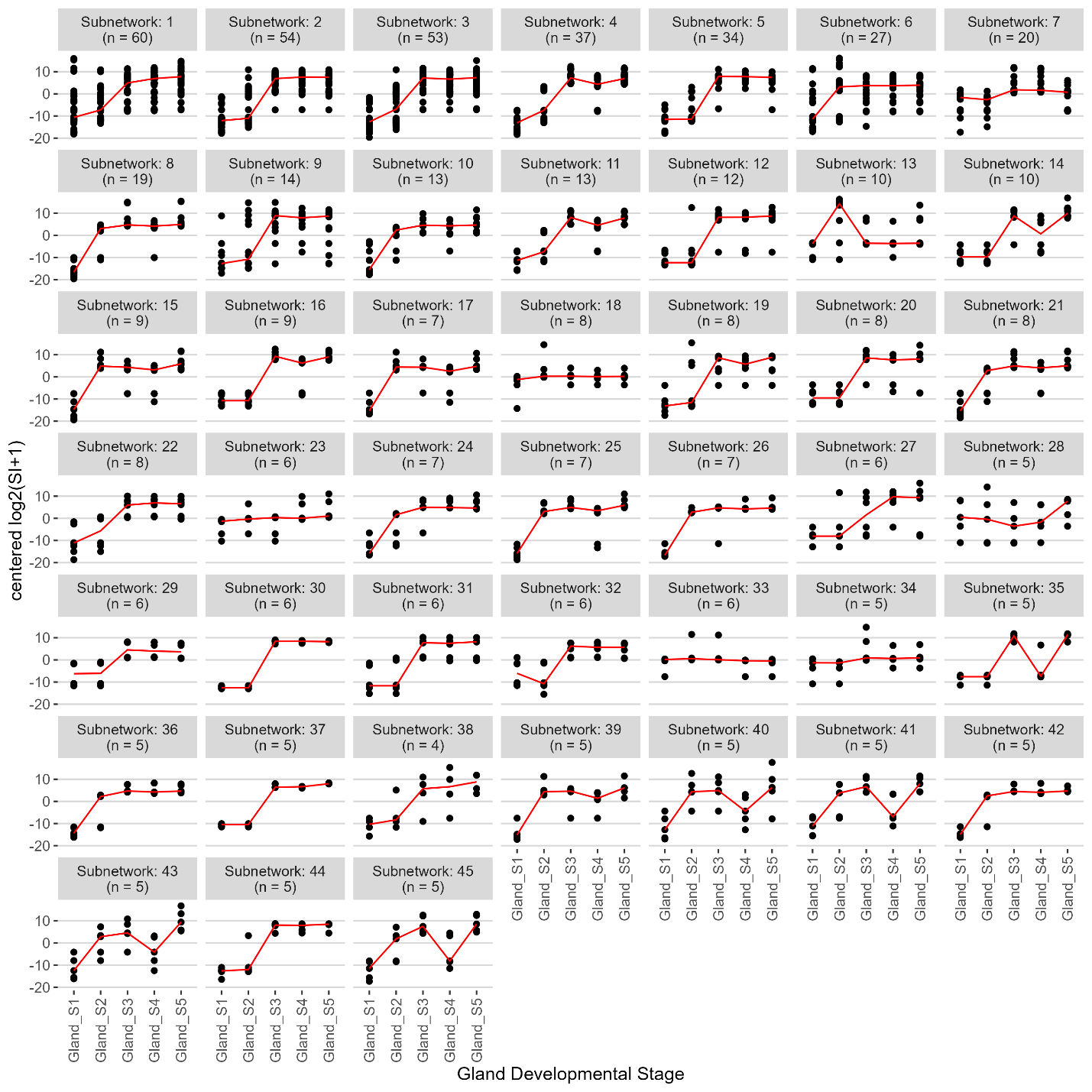


Supplementary Fig. 33. Non-volatile subnetwork analysis of hop cone development. Feature-based molecular networking (FBMN) of the non-volatile chemical information gathered throughout hop cone development was utilized to isolate 45 subnetworks that contained at least five chemical features. For each subnetwork, centered log_2_(*x*+1) transformed signal intensity information of the respective chemical features (total number per subnetwork displayed in header as ‘n’) were plotted for all five developmental stages of the gland fraction after averaging the replicates and removal of bract fraction-specific features. The red line connects the median values of all data points per developmental stage. Source data are provided as a Source Data file.


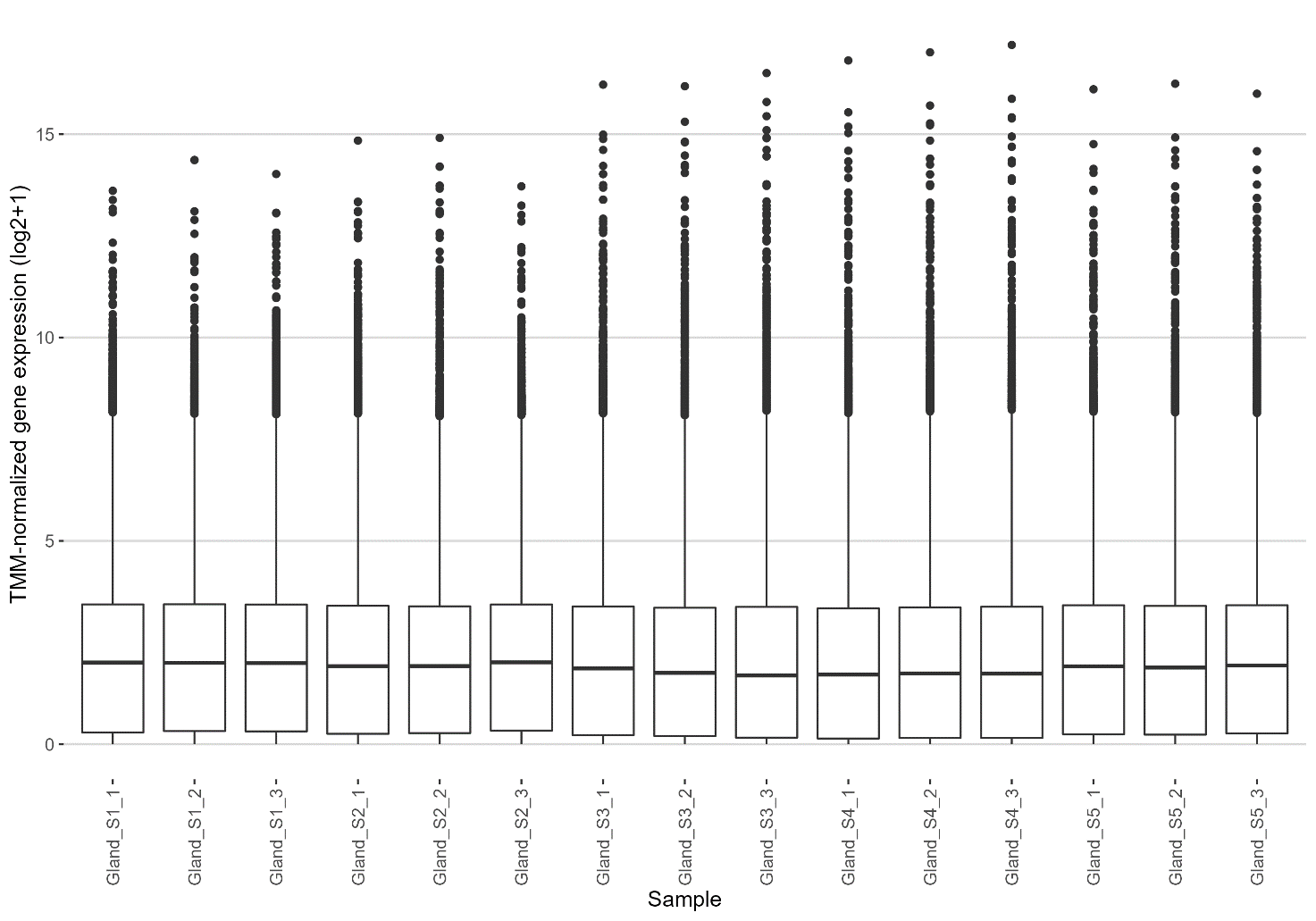


Supplementary Fig. 34. Cross-normalized gene expression count data distribution for mRNA reads throughout hop cone development. Boxplots were generated based on TMM-normalized gene expression data derived from the glandular fraction sample set comprising five stages of hop cone development (S1-S5). Genes had to be expressed in at least one sample, which resulted in total 46977 genes being represented by each displayed boxplot. Before plotting, the displayed gene count data was transformed to a base-2 logarithm with a pseudo count of +1. Boxplots were generated using the default settings of the ggplot2 package in R. The centre line indicates the median. The box limits represent the 25^th^ and 75^th^ percentiles (interquartile range, IQR). Whiskers extend to the smallest and largest observations within 1.5 × IQR of the lower and upper quartiles. Observations beyond the whiskers are plotted as individual outliers. Source data are provided as a Source Data file.


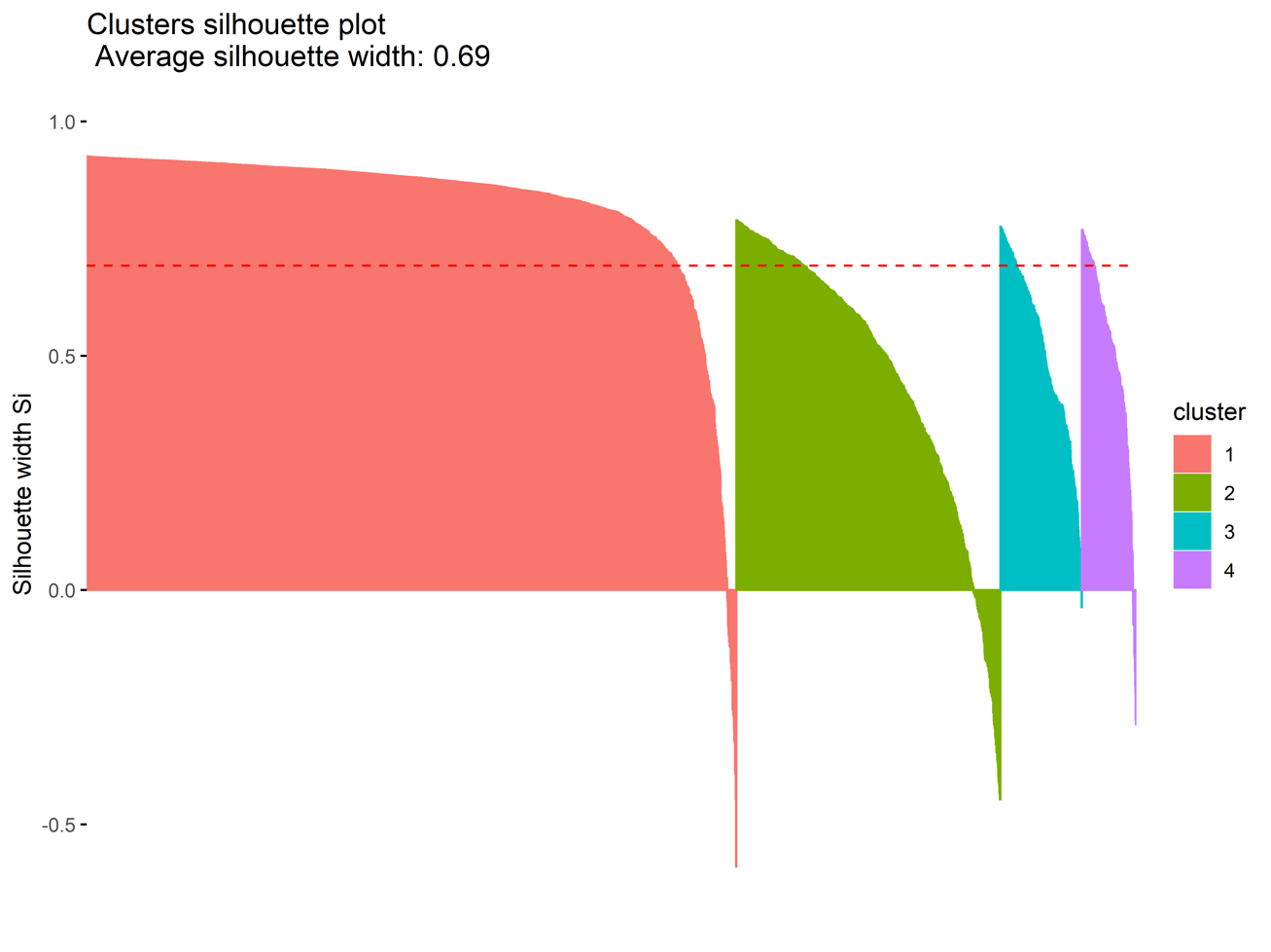


Supplementary Fig. 35. Silhouette plot for DEG cluster quality control. The silhouette plot summarizes the silhouette analysis conducted on four differentially expressed gene (DEG) clusters that were generated based on gene expression data derived from glandular fractions collected during hop cone development. Silhouette value indicates how similar an object is to its own cluster compared to other clusters, with a given range between −1 and 1, plotted here as y-axis for each DEG found on the x-axis. Here, high silhouette values indicate a good match of the gene to its respective cluster, while being poorly matched to neighboring clusters. DEG with negative silhouette values were removed from the set for downstream analysis. The red dotted line highlights the average silhouette value of 0.69. Source data are provided as a Source Data file.


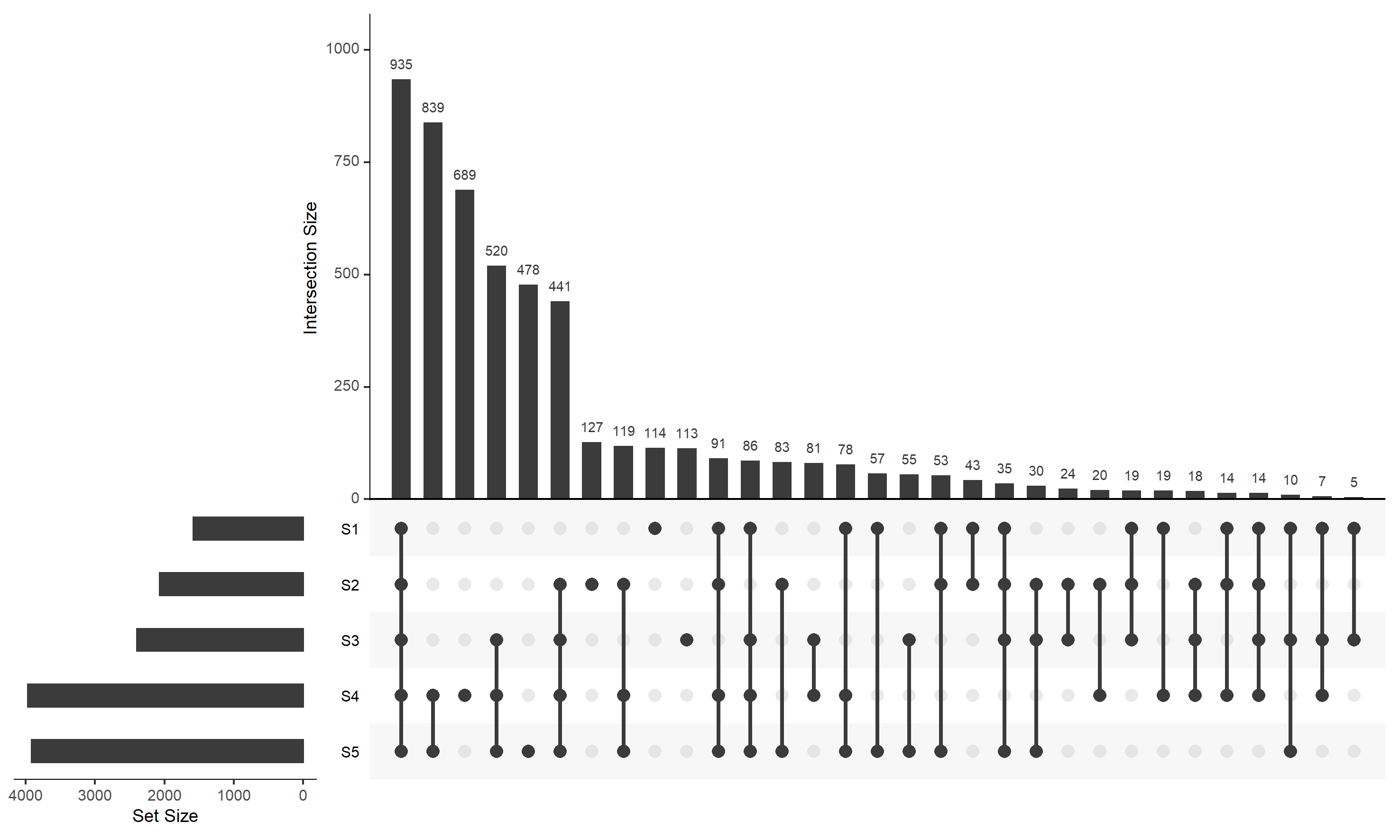


Supplementary Fig. 36. UpSet plot of allelic expression between ortholog gene pairs during hop cone development. For a stage-resolved insight into allele specific expression (ASE) throughout hop cone development, a gene expression set of 19725 ortholog gene pairs was utilized within a stage-separated differential expression analysis. Total amount of gene pairs found with significant ASE (adjusted *P* < 0.05) are displayed as bars on the left giving the set size for each stage. Intersections of gene pairs that showed significant ASE were visualized for all possible stage combinations as shown below, while the total size of each intersection is shown on top of the UpSet plot. All combinations were sorted by size in decreasing order from left to right. Source data are provided as a Source Data file.

| **Supplementary Table 1. PacBio HiFi sequencing statistics of cv. Apollo.** | | | |  |
| --- | --- | --- | --- | --- |
|  | | | | |
| **Smart cell** | **Sub reads** | **Hifi reads** | **Bases (Gb)** | **Mean read length (kb)** |
| **m64036_201209_173153** | 2 573 550 | 842 096 | 20.7849 | 24.68 |
| **m64036_210115_180136** | 3 131 587 | 941 191 | 22.6993 | 24.12 |
| **m64036_210209_133642** | 2 337 373 | 815 165 | 16.6805 | 20.46 |
| **m64036_210226_172459** | 4 312 508 | 1 439 282 | 29.5313 | 20.52 |
| **m64036_210227_234015** | 5 263 867 | 1 593 444 | 32.4444 | 20.36 |
| **Total** | 17 618 885 | 5 631 178 | 122.1405 | 22.03 |

This table summarizes the number of reads, data size and mean read length obtained from each PacBio HiFi sequencing run.

**Supplementary Table 2. Assembly statistics for Apollo and Cascade genomes.**

| **Cultivar** | **Apollo** | **Cascade** |
| --- | --- | --- |
| **Pseudomolecule statistics** | | |
| Total scaffolds in pseudomolecule | 48 | 1,583 |
| Pseudomolecule size (Gb) | 2.62 | 3.71 |
| N50 of assembled assembly (Mb) | 272.39 | 345.3 |
| Assembly per ps | 38 | 8,172 |
| **Contig statistics** | | |
| Total contigs | 797 | 8,661 |
| Assembly size (Gb) | 8.39 | 8.71 |
| N50 (bp) | 361,592,828 | 672,608 |
| **BUSCO** | | |
| Complete BUSCOs (C) | 1580 (97.5%) | 1549 (96%) |
| Complete and single-copy BUSCOs (S) | 1498 (92.3%) | 1495 (92.6%) |
| Complete and duplicated BUSCOs (D) | 82 (5.06) | 55 (3.4) |
| Fragmented BUSCOs (F) | 8 (0.5) | 23 (1.4) |
| Missing BUSCOs (M) | 26 (1.6) | 42 (2.6) |
| Total BUSCO groups searched | 1614 | 1614 |

Table summarizing statistics of the haploid assemblies *Humulus* cvs. Apollo (HA, this study) and Cascade.

| **Supplementary Table 3. Pseudomolecule wise distribution of high confident genes identified in the haploid assembly of hop cv. Apollo.** | |
| --- | --- |
|  | |
| **Pseudomolecules** | **Genes** |
| **chr1** | 2352 |
| **chr2** | 1631 |
| **chr3** | 1257 |
| **chr4** | 1891 |
| **chr5** | 1378 |
| **chr6** | 1262 |
| **chr7** | 1126 |
| **chr8** | 1555 |
| **chr9** | 2135 |
| **chrX** | 1171 |
| **Total** | 15758 |

This table provides a summary of the number of high confident genes annotated from each chromosome in the haploid assembly of hop cv. Apollo.

| **Supplementary Table 4. Summary of gene based BUSCO (v5.3.2, eudicots_odb10) completeness assessment for the haplotype resolved genome assembly (contigs) of hop cv. Apollo.** | | | |
| --- | --- | --- | --- |
|  | | | |
|  |  |  |  |
| **BUSCO class** | **Both phases (%)** | **Phase 1 (%)** | **Phase 2 (%)** |
| Complete single-copy | 102 (4.39) | 2120 (91.14) | 2108 (90.63) |
| Complete duplicated | 2145 (92.22) | 85 (3.65) | 61 (2.62) |
| fragmented | 12 (0.52) | 30 (1.29) | 30 (1.29) |
| missing | 67 (2.88) | 91 (3.91) | 127 (5.46) |
| Total searched | 2326 | 2326 | 2326 |
|  |  |  |  |
| This table reports the proportions of complete (single-copy and duplicated), fragmented, and missing BUSCOs identified from combined and individual phased assemblies of hop cv. Apollo. The table provides an overall measure of assembly and annotation quality. Only primary proteins were considered for the analysis. | | | |

**Supplementary Table 5. Summary statistics of chromosome scale haplotype resolved phased assembly of hop cv. Apollo.**

|  | **Phase1** | **Phase2** | **Combined** | **Unassembled** | **Unassembled (Mb)** |
| --- | --- | --- | --- | --- | --- |
| **Contig statistics (hifiasm)** |  |  |  |  |  |
| No. of contigs | 999 | 528 | 1527 | - |  |
| N50 (Mb) | 128 | 136.58 | 128 | - |  |
| Maximum length (Mb) | 188.19 | 242.34 | 242.34 | - |  |
| Average length (Mb) | 2.25 | 5.57 | 3.4 | - |  |
| Total size (Mb) | 2247.45 | 2939.92 | 5187.37 | - |  |
|  |  |  |  |  |  |
| **Contig statistics (AllHiC)** |  |  |  |  |  |
| No. of contigs | 57 | 54 |  | 1416 |  |
| N50 (Mb) | 174.77 | 108.44 |  | 0.07 |  |
| Maximum length (Mb) | 242.34 | 191.35 |  | 2.27 |  |
| Average length (Mb) | 44.85 | 47.02 |  | 0.06 |  |
| Total size (Gb) | 2.56 | 2.54 |  | 0.09 | 91.64 |
|  |  |  |  |  |  |
| **Contig statistics (TRITEX)** |  |  |  |  |  |
| No. of contigs | 44 | 37 |  |  |  |
| N50 (Mb) | 121.06 | 108.44 |  |  |  |
| Maximum length (Mb) | 232 | 191.35 |  |  |  |
| Average length (Mb) | 59.53 | 66.88 |  |  |  |
| Total size (Gb) | 2.62 | 2.47 |  | 0.002 | 2.07 |
|  |  |  |  |  |  |
| **Pseudomolecule statistics** |  |  |  |  |  |
| Number of Pseudomolecules | 10 | 10 |  |  |  |
| Pseudomolecule size (Gb) | 2.62 | 2.47 |  |  |  |
| Longest Pseudomolecule (Mb) | 335.79 | 308.53 |  |  |  |
|  |  |  |  |  |  |
| **BUSCO (v5.3.2, eudicots_odb10)*** | number (%) | number (%) |  |  |  |
| Number of Complete BUSCOs (C) | 1588 (98.39 ) | 1573 (97.46 ) |  |  |  |
| number of Complete and single-copy BUSCOs (S) | 1550 (96.03 ) | 1541 (95.48 ) |  |  |  |
| Number of Complete and duplicated BUSCOs (D) | 38 (2.35 ) | 32 (1.98 ) |  |  |  |
| Number of Fragmented BUSCOs (F) | 9 (0.56 ) | 10 (0.62 ) |  |  |  |
| Number of Missing BUSCOs (M) | 17 (1.05 ) | 31 (1.92 ) |  |  |  |
| Total BUSCO groups searched | 1614 (100 ) | 1614 (100 ) |  |  |  |

This table summarizes the results from individual stages in construction of chromosome scale haplotype resolved genome assembly of hop cv. Apollo.

**Supplementary Table 6. Pseudomolecule wise distribution of SNPs with phase switch errors.**

| **Pseudomolecules** | **Total SNPs** | **SNPs with phase switch** | **Proportion** |
| --- | --- | --- | --- |
| **chr01** | 401747 | 23470 | 0,058 |
| **chr02** | 9018 | 1839 | 0,204 |
| **chr03** | 907890 | 2282 | 0,003 |
| **chr04** | 1059502 | 4453 | 0,004 |
| **chr05** | 822702 | 1087 | 0,001 |
| **chr06** | 738298 | 781 | 0,001 |
| **chr07** | 181243 | 67676 | 0,373 |
| **chr08** | 68733 | 938 | 0,014 |
| **chr09** | 922313 | 17913 | 0,019 |
| **chrX** | 774599 | 2314 | 0,003 |
| **Total** | 5886045 | 122753 | 0,021 |
| **% of phase switch** | 2,085 |  |  |

This table summarizes the results of phase switch error analysis conducted to determine the quality of haplotype resolved phased assembly of hop cv. Apollo.

**Supplementary Table 7. Pseudomolecule wise distribution of SNPs identified from different bi-parental mapping populations.**

|  | **Populations** | | | | | | | | | | | |
| --- | --- | --- | --- | --- | --- | --- | --- | --- | --- | --- | --- | --- |
|  | **Apollo × PubM_740** | | | | **Cascade × HL-19HL-19-060-002M** | | | | **Zenith × USDA21058M** | | | |
| **Pseudomolecules** | **SNPs identified** | **Apollo** | **PubM_740** | **Total (selected)** | **SNPs identified** | **Cascade** | **HL-19HL-19-060-002M** | **Total (selected)** | **SNPs identified** | **Zenith** | **USDA21058M** | **Total (selected)** |
| **chr01** | 2576550 | 466960 | 28985 | 495945 | 6699 | 1782 | 212 | 1994 | 19343 | 137 | 286 | 423 |
| **chr02** | 1346798 | 9472 | 183600 | 193072 | 6698 | 1408 | 157 | 1565 | 19882 | 188 | 233 | 421 |
| **chr03** | 4293039 | 1274489 | 66786 | 1341275 | 5824 | 1176 | 113 | 1289 | 19159 | 87 | 1286 | 1373 |
| **chr04** | 5424322 | 1567047 | 54358 | 1621405 | 7616 | 1477 | 126 | 1603 | 24926 | 153 | 2290 | 2443 |
| **chr05** | 4129767 | 1115419 | 83455 | 1198874 | 6249 | 1117 | 166 | 1283 | 24210 | 187 | 376 | 563 |
| **chr06** | 3466206 | 1100569 | 11388 | 1111957 | 5724 | 1014 | 108 | 1122 | 18111 | 135 | 386 | 521 |
| **chr07** | 951888 | 79489 | 13961 | 93450 | 4267 | 107 | 102 | 209 | 18452 | 110 | 225 | 335 |
| **chr08** | 6173376 | 30713 | 1396535 | 1427248 | 6277 | 1225 | 200 | 1425 | 14143 | 53 | 1339 | 1392 |
| **chr09** | 4995440 | 1358480 | 92577 | 1451057 | 6988 | 505 | 256 | 761 | 25101 | 145 | 1479 | 1624 |
| **chrX** | 2422544 | 13630 | 1948 | 15578 | 4978 | 202 | 13 | 215 | 9793 | 75 | 95 | 170 |
| **Total** | 35779930 | 7016268 | 1933593 | 8949861 | 61320 | 10013 | 1453 | 11466 | 193120 | 1270 | 7995 | 9265 |
| **Maximum** | 6173376 | 1567047 | 1396535 | 1621405 | 7616 | 1782 | 256 | 1994 | 25101 | 188 | 2290 | 2443 |
| **Minimum** | 951888 | 9472 | 1948 | 15578 | 4267 | 107 | 13 | 209 | 9793 | 53 | 95 | 170 |

This table summarizes the results of variant (SNP) calling for different bi-parental mapping populations aligned to phase2 of haplotype resolved genome assembly of hop cv. Apollo.

**Supplementary Table 8. Summary of linkage-mapping results obtained using the pseudo-testcross strategy across multiple bi-parental populations.**

|  |  |  |  |  |  |  | **Accessions** |  |  |  |  |  |  |
| --- | --- | --- | --- | --- | --- | --- | --- | --- | --- | --- | --- | --- | --- |
|  |  | **Cascade** | | | | | | **Apollo** | | | **USDA21058M** | | |
| **Pseudomolecules** | **Chromosome Length (Mb)** | **Total Markers** | **Markers used for linkage map** | **Percentage** | **Map length (cM)** | **Marker density (Markers/cM)** | **Recombination rate (cM/Mb)** | **Total Markers** | **Markers used for linkage map** | **Percentage** | **Total Markers** | **Markers used for linkage map** | **Percentage** |
| **chr01** | 188.17 | 1782 | 1616 | 90.68 | 350.7996765 | 4.60662 | 1.86 | 1000 | 946 | 94.6 | 286 | 114 | 39.86 |
| **chr02** | 212.78 | 1408 | 1272 | 90.34 | 304.0599619 | 4.18339 | 1.43 | 1000 | 174 | 17.4 | 233 | 60 | 25.75 |
| **chr03** | 246.39 | 1176 | 1051 | 89.37 | 544.9357431 | 1.92867 | 2.21 | 1000 | 980 | 98 | 1286 | 943 | 73.33 |
| **chr04** | 308.53 | 1477 | 1376 | 93.16 | 340.9680141 | 4.03557 | 1.11 | 1000 | 958 | 95.8 | 2290 | 1478 | 64.54 |
| **chr05** | 244.76 | 1117 | 906 | 81.11 | 413.1791271 | 2.19275 | 1.69 | 1000 | 987 | 98.7 | 376 | 133 | 35.37 |
| **chr06** | 222.94 | 1014 | 935 | 92.21 | 429.263855 | 2.17815 | 1.93 | 1000 | 951 | 95.1 | 386 | 103 | 26.68 |
| **chr07** | 186.55 | 107 | 47 | 43.93 | 18.16390222 | 2.58755 | 0.1 | 1000 | 666 | 66.6 | 225 | 42 | 18.67 |
| **chr08** | 301.44 | 1225 | 1106 | 90.29 | 341.7867067 | 3.23594 | 1.13 | 1000 | 189 | 18.9 | 1339 | 927 | 69.23 |
| **chr09** | 299.35 | 505 | 275 | 54.46 | 110.3276058 | 2.49258 | 0.37 | 1000 | 963 | 96.3 | 1479 | 485 | 32.79 |
| **chrX** | 263.52 | 202 | 167 | 82.67 | 11.20548703 | 14.9034 | 0.04 | 1000 | 683 | 68.3 | 95 | 16 | 16.84 |
| **Total** | 2474.43 | 10013 | 8751 |  | 2864.690079 |  |  | 10000 | 7497 |  | 7995 | 4301 |  |
| **Average** |  |  |  | 80.822 |  | 4.234 | 1.187 |  |  | 74.97 |  |  | 40.306 |

This table summarizes the results of linkage mapping analysis carried out following pseudo-test cross strategy in different bi-parental populations.

**Supplementary Table 9. Chromosome wise distribution of SNPs used for population genomic study using diverse hop accessions.**

| **Chromosome** | **SNPs** | **Used for analysis** |
| --- | --- | --- |
| chr01 | 7397 | 4027 |
| chr02 | 6068 | 3331 |
| chr03 | 5051 | 2892 |
| chr04 | 7686 | 4341 |
| chr05 | 6077 | 3439 |
| chr06 | 5549 | 3054 |
| chr07 | 4667 | 2546 |
| chr08 | 5046 | 2846 |
| chr09 | 8141 | 4404 |
| chr10 | 4088 | 2298 |
| Total | 59770 | 33178 |

This table summarizes the results of variant (SNP) calling analysis conducted using the diverse hop accessions.

| **Supplementary Table 10. Summery statistic of Fst analysis.** | | |
| --- | --- | --- |
|  | | |
| **POP1** | **POP2** | **Fst** |
| lupuloides | Cultivar | 0.34 |
| lupulus | Cultivar | 0.2 |
| lupulus_ME | Cultivar | 0.25 |
| neomexicanus | Cultivar | 0.42 |
| lupulus | lupuloides | 0.71 |
| lupulus_ME | lupuloides | 0.7 |
| **neomexicanus** | **lupuloides** | **0.27** |
| **lupulus_ME** | **lupulus** | **0.31** |
| neomexicanus | lupulus | 0.8 |
| neomexicanus | lupulus_ME | 0.78 |

This table summarizes the results of Fst analysis conducted between the different hop sub-species.

**Supplementary Table 11. Genome composition of hop and hemp.**

| **Mb** | **Hemp** | **Hop phase 1** | **Hop phase 2** | **Hop  lupoloides  ancestry** | **Hop  lupulus  ancestry** | **Hop  phase 1/Hemp** | **phase1/phase2** | **North  American/European** |
| --- | --- | --- | --- | --- | --- | --- | --- | --- |
| Transposon | 492.54 | 2185.07 | 2049.09 | 2120.42 | 2113.74 | *4.4* | *1.1* | *1.0* |
| **Retrotransposon** | **429.73** | **1924.23** | **1793.82** | **1884.51** | **1833.54** | ***4.5*** | ***1.1*** | ***1.0*** |
| LTR-retrotransposon | 426.96 | 1920.58 | 1790.26 | 1881.05 | 1829.79 | *4.5* | *1.1* | *1.0* |
| Copia | 157.79 | 232.78 | 229.80 | 225.10 | 237.48 | *1.5* | *1.0* | *0.9* |
| Gypsy | 93.19 | 1428.12 | 1325.58 | 1400.83 | 1352.87 | *15.3* | *1.1* | *1.0* |
| unknown | 175.98 | 259.69 | 234.88 | 255.13 | 239.44 | *1.5* | *1.1* | *1.1* |
| non LTR-retrotransposon | 2.77 | 3.64 | 3.56 | 3.46 | 3.75 | *1.3* | *1.0* | *0.9* |
| LINE | 2.77 | 3.48 | 3.39 | 3.29 | 3.57 | *1.3* | *1.0* | *0.9* |
| unknown | 0.00 | 0.17 | 0.18 | 0.17 | 0.17 |  | *0.9* | *1.0* |
| **DNA transposon** | **62.80** | **260.84** | **255.27** | **235.91** | **280.20** | ***4.2*** | ***1.0*** | ***0.8*** |
| TIR | 47.86 | 235.77 | 232.44 | 211.64 | 256.57 | *4.9* | *1.0* | *0.8* |
| CACTA | 10.04 | 95.78 | 93.30 | 91.50 | 97.57 | *9.5* | *1.0* | *0.9* |
| Mutator | 25.03 | 91.77 | 91.57 | 88.60 | 94.73 | *3.7* | *1.0* | *0.9* |
| PIF_Harbinger | 5.49 | 6.74 | 6.87 | 6.66 | 6.95 | *1.2* | *1.0* | *1.0* |
| Tc1_Mariner | 0.26 | 20.21 | 20.01 | 4.45 | 35.77 | *77.0* | *1.0* | *0.1* |
| hAT | 7.05 | 21.28 | 20.70 | 20.43 | 21.55 | *3.0* | *1.0* | *0.9* |
| helitron | 14.94 | 25.07 | 22.83 | 24.27 | 23.63 | *1.7* | *1.1* | *1.0* |
| unknown | 28.48 | 89.07 | 85.52 | 86.68 | 87.91 | *3.1* | *1.0* | *1.0* |
| **Repeats total Mb** | **521.02** | **2274.14** | **2134.61** | **2207.10** | **2201.65** | ***4.4*** | *1.1* | *1.0* |
|  |  |  |  |  |  |  |  |  |
| **Genes number** | **25261.00** | **29915.00** | **29371.00** | **29394.00** | **29892.00** | ***1.2*** | ***1.0*** | ***1.0*** |
| Genes Mb | 79.23 | 88.64 | 86.55 | 86.58 | 88.61 | *1.1* | *1.0* | *1.0* |
| CDS exons Mb | 33.22 | 35.79 | 35.11 | 35.31 | 35.60 | *1.1* | *1.0* | *1.0* |
| CDS introns Mb | 46.00 | 52.85 | 51.44 | 51.28 | 53.01 | *1.1* | *1.0* | *1.0* |
| **Assembly size Mb** | **876.15** | **2619.24** | **2474.42** | **2541.47** | **2552.19** | ***3.0*** | ***1.1*** | ***1.0*** |
|  |  |  |  |  |  |  |  |  |
| Repeats % of assembly | 59.47 | 86.82 | 86.27 | 86.84 | 86.27 | *1.5* | *1.0* | *1.0* |
| Genes % of assembly | 9.04 | 3.38 | 3.50 | 3.41 | 3.47 | *0.4* | *1.0* | *1.0* |
| unassigned % of assembly | 31.49 | 9.79 | 10.24 | 9.75 | 10.26 | *0.3* | *1.0* | *0.9* |

Detailed overview on genome composition of tested hop and hemp genomes.

**Supplementary Table 12. Ancestral phase admixture.**

|  | **Mb** | **Percent of assembly phase 1 or 2** | **Percent of assembly** | **Number of genes** | **Percent of genes** | **Number of fl-LTRs** | **Percent of fl-LTRs** |
| --- | --- | --- | --- | --- | --- | --- | --- |
| **Assembly phase 1** | **2619** | **100,0** | **51,4** | 29915 | 50,5 | 28141 | 54,7 |
| North American origin | 2152 | 82,2 | 42,2 | 23376 | 39,4 | 23968 | 46,6 |
| European origin | 467 | 17,8 | 9,2 | 6539 | 11,0 | 4173 | 8,1 |
| **Assembly phase 2** | **2474** | **100,0** | **48,6** | 29371 | 49,5 | 23331 | 45,3 |
| European origin | 2085 | 84,3 | 40,9 | 23353 | 39,4 | 19094 | 37,1 |
| North American origin | 390 | 15,7 | 7,6 | 6018 | 10,2 | 4237 | 8,2 |
|  |  |  |  |  |  |  |  |
| North American origin | 2541 |  | 49,9 | 29394 | 49,6 | 28205 | 54,8 |
| European origin | 2552 |  | 50,1 | 29892 | 50,4 | 23267 | 45,2 |
|  |  |  |  |  |  |  |  |
| Total | 5094 |  | 100,0 | 59286 | 100,0 | 51472 | 100,0 |

Distribution of ancestral origin across the two assembly phases for assembly size, gene number and number of full length LTR-retrotransposons.

**Supplementary Table 13. Summary of structural variations identified between homologous chromosomes from the haplotype resolved chromosome scale genome assembly of hop cv. Apollo.**

|  | **Pseudo- molecules** | **chr01** | **chr02** | **chr03** | **chr04** | **chr05** | **chr06** | **chr07** | **chr08** | **chr09** | **chrX** | **Total** |
| --- | --- | --- | --- | --- | --- | --- | --- | --- | --- | --- | --- | --- |
| **Chromosome  length** | **Phase1 (Mb)** | 201.5 | 211.94 | 282.74 | 335.79 | 272.18 | 234.99 | 185.83 | 297.53 | 324.22 | 272.52 | 2619.24 |
|  | **Phase2 (Mb)** | 188.17 | 212.78 | 246.39 | 308.53 | 244.76 | 222.94 | 186.55 | 301.44 | 299.35 | 263.52 | 2474.43 |
|  | **Difference (Mb)** | 13.33 | -0.84 | 36.35 | 27.26 | 27.42 | 12.05 | -0.72 | -3.91 | 24.87 | 9 | 144.81 |
| **Gene  models** | **Phase1** | 4064 | 2803 | 2743 | 3675 | 2665 | 2764 | 2065 | 3188 | 4357 | 2596 | 30920 |
|  | **Phase2** | 3867 | 2808 | 2608 | 3798 | 2838 | 2843 | 2047 | 3007 | 4074 | 2508 | 30398 |
| **Structural  variations  (SVs)** | **Presence** | 4272 | 66 | 6813 | 9130 | 7286 | 5454 | 783 | 1410 | 9106 | 8273 | 44320 |
|  | **Absence** | 3543 | 35 | 5635 | 8005 | 5934 | 3536 | 895 | 1132 | 7896 | 5379 | 36611 |
|  | **Translocations** | 269 | 3 | 562 | 879 | 524 | 314 | 117 | 261 | 547 | 962 | 3476 |
|  | **Inversions** | 15 | 1 | 48 | 31 | 33 | 24 | 2 | 5 | 27 | 29 | 186 |
| **Presence-absence  variations (PAVs)** | **Phase1 (Mb)** | 165.89 | 3.01 | 197.86 | 266.76 | 207.32 | 120.23 | 13.52 | 30.3 | 276.63 | 188.06 | 1469.58 |
|  | **Phase2 (Mb)** | 152.6 | 3.9 | 167.89 | 242.86 | 185.36 | 116.84 | 14.71 | 34.87 | 253.31 | 181.8 | 1354.14 |
| **Translocations** | **Phase1 (Mb)** | 1.82 | 0.06 | 4.54 | 6.74 | 4.98 | 3.28 | 1.37 | 2.67 | 3.66 | 8.11 | 37.23 |
|  | **Phase2 (Mb)** | 1.81 | 0.06 | 4.49 | 6.65 | 4.9 | 3.24 | 1.39 | 2.72 | 3.65 | 8.14 | 37.05 |
| **Inversions** | **Phase1 (Mb)** | 8.37 | 4.74 | 57.91 | 31.44 | 32.58 | 94.83 | 4.75 | 1.87 | 8.47 | 61.05 | 306.01 |
|  | **Phase2 (Mb)** | 8.4 | 4.58 | 52.18 | 28.16 | 27.64 | 86.31 | 4.49 | 0.86 | 6.97 | 59.19 | 278.78 |
| **Total (Mb)** | **Phase1 (Mb)** | 176.08 | 7.81 | 260.31 | 304.94 | 244.88 | 218.34 | 19.64 | 34.84 | 288.76 | 257.22 | 1812.82 |
|  | **Phase2 (Mb)** | 162.81 | 8.54 | 224.56 | 277.67 | 217.9 | 206.39 | 20.59 | 38.45 | 263.93 | 249.13 | 1669.97 |
| **Total (%)** | **Phase1 (Mb)** | 87.38 | 3.69 | 92.07 | 90.81 | 89.97 | 92.91 | 10.57 | 11.71 | 89.06 | 94.39 | 69.21 |
|  | **Phase2 (Mb)** | 86.52 | 4.01 | 91.14 | 90 | 89.03 | 92.58 | 11.04 | 12.76 | 88.17 | 94.54 | 67.49 |

This table summarizes the results of structural variation analysis conducted using the two phases of haplotype resolved chromosome scale genome assembly of hop cv. Apollo.

**Supplementary Table 14. Pedigree of hop accessions tested for the presence of a ~85 Mb inversion observed on phase 1 of chr06 in haplotype resolved phased genome assembly of cv. Apollo.**

| **Cultivar** | **Relation to Brewer's Gold or lines in the Brewer's Gold lineage** | **PCR result** | **Coverage plot of GBS data mapped to Apollo assemby available** | **phase 1 chromosome 6 present (coverage plot)** | **Pedigree** | **Reference** | **Match** |
| --- | --- | --- | --- | --- | --- | --- | --- |
| Brewer's  Gold |  | + | yes | yes | BB1 (Manitoba wild) X Open-pollination | *20 | Brewers_Gold |
| Bullion | Sister to Brewer's Gold | - | no | n/a | BB1 (Manitoba wild) X Open-pollination | *29 | #N/A |
| Cascade | Not related to Brewer's Gold | - | yes | yes | (Fuggle X (Serebrjanka × seedling of Fuggle)) X open-pollinated | *20 | Cascade |
| Comet | Not related to Brewer's Gold | - | yes | yes | Sunshine seedling × Utah wild male | *20 | Comet |
| Saaz | Not related to Brewer's Gold | - | yes | no | European landrace | *20 | Saaz |
| Eroica | Daugther of Brewer's Gold | + | yes | yes | Brewer's Gold X Open-pollination | *20 | Eroica |
| Galena | Daugther of Brewer's Gold | + | yes | yes | Brewer's Gold X Open-pollination | *20 | Galena |
| Horizon | Daugther of Nugget | + | yes | yes | Nugget X 64035M | *20 | Horizon |
| Triumph | Daugther of Nugget | + | no | n/a | Nugget X USDA 2110M | *30 | #N/A |
| Centennial | Daugther of 63015M | + | yes | yes | OR6619-04 X USDA63015M | *20 | Centennial |
| Chinook | Brewer's Gold in pedigree | - | yes | yes | Petham Golding X USDA63012M (Brewer‘s Gold X utah wild hop) | *20 | Chinook |
| Olympic | Brewer's Gold in pedigree | - | yes | yes | 6619-04 X 64028M (Brewer's Gold2 x Fuggle-Fuggle S) x (Brewer's Gold2 x East Kent Golding - Bavarian Seedling) | *31 | Olympic |
| Crystal | Brewer's Gold in pedigree | - | yes | yes | [USDA 21397 x USDA 21381M; Tetraploid Hallertauer Mittelfrueh x (Cascade x USDA 65009-64034M)](https://www.ars.usda.gov/ARSUserFiles/2450/hopcultivars/21397.html) | *32 | Crystal |
| Glacier | Brewer's Gold in pedigree | + | yes | yes | Strisselspalt X US male (31.2% Brewer‘s Gold, 25% Northern Brewer,43.8% English and German hops) | *20 | Glacier |
| Tahoma | Brewer's Gold in pedigree. | + | no | n/a | Daugther of Glacier | n/a | #N/A |
| Citra | Brewer's Gold in pedigree. | + | no | n/a | Hallertauer Mtf. X (US Tettnang X Brewer's Gold X seedling of east Kent Golding) | *20 | #N/A |
| Apollo | Brewer's Gold in pedigree. | + | yes | yes | Zeus x (98001 x USDA19058M) | *20 | Apollo |
| Zeus | Brewer's Gold in pedigree. | + | yes | yes | USDA65009 (Brewer's Gold x Early Green Golding) x unknown male | *20 | Zeus_1 |
| Mpub700 | Brewer's Gold in pedigree. | - | no | n/a | (Galena x Nugget seedling) x (M19058) | n/a | #N/A |
| Nugget | Brewer's Gold in pedigree | + | yes | yes | USDA65009 (Brewer's Gold x Early Green Golding) x USDA63015M (Brewer's Gold x East Kent Golding) | *20 | Nugget |
| M63015 | Son of Brewer's Gold | + | yes | yes | Brewer’s Gold//East Kent Golding/Bavarian-seedling | *33 | USDA_63015m |
| M19058 | Not related to Brewer's Gold | - | yes | yes | Early Green x Open-pollination | *21 | USDA_19058m_1 |
| East Kent  Golding | Not related to Brewer's Gold | - | yes | no | no information | n/a | Kent_Golding |

| **Supplementary Table 15. Results of ANOVA analysis conducted to study difference in α acid content between the clusters identified from GWAS.** | | | | |
| --- | --- | --- | --- | --- |
|  | | | | |
|  | **diff** | **lower** | **upper** | **alpha_acid** |
| medium-low | 0.24939522 | 0.06866289 | 0.4301276 | 0.0038975 |
| high-low | 0.34133066 | 0.1437073 | 0.538954 | 0.0002178 |
| high-medium | 0.09193544 | -0.06794973 | 0.2518206 | 0.3631196 |

This table summarizes the results of ANOVA analysis conducted to evaluate differences in alpha-acid content among clusters identified in the k-mer analysis.

**Supplementary Table 16. Parameters used for raw GC-DHS-MS spectral data processing in MzMine2.** Detailed list of modules and corresponding parameters that have been applied using MZmine2 (v2.53) to process raw spectral data coming from the utilized GC-DHS-MS system.

| **Step** | **Parameter** | **Value** |
| --- | --- | --- |
| Mass Detection | mass detector | centroid |
|  | polarity | + |
|  | MS level | 1 |
|  | noise level | 1000 |
|  | retention time | full range |
| ADAP Chromatogram Builder | MS level | 1 |
|  | polarity | + |
|  | spectrum type | centroid |
|  | min group size | 3 |
|  | retention time | full range |
|  | min highest intensity | 1000 |
|  | group intensity threshold | 1000 |
|  | *m/z* tolerance | 0.1 *m/z* (5 ppm) |
| Chromatogram Deconvolution | algorithm | Local Min Search |
|  | chromatographic threshold | 95% |
|  | min in RT range | 0.01 min |
|  | min relative height | 1% |
|  | min absolute height | 5000 |
|  | min ratio of peak top/edge | 1.2 |
|  | peak duration range | 0.0 - 1 min |
|  | *m/z* center calculation | median |
| ADAP Alignment | *m/z* tolerance | 0.1 *m/z* (10 ppm) |
|  | RT tolerance | 0.1 min |
|  | Score weight | 0.1 |
|  | Score threshold | 0.75 |
|  | RT time similarity | RT Difference |
| Local spectra database search | MS level | 1 |
|  | precursor *m/z* tolerance | 0.1 *m/z* (5 ppm) |
|  | minimum ion intensity | 0 |
|  | spectral *m/z* tolerance | 0.2 *m/z* (10 ppm) |
|  | RT tolerance | 0.1 min |
|  | min matched signals | 4 |
|  | similarity | weighted dot-product cosine |
|  | weights | NONE |
|  | min cosine similarity | 0.7 |
| MSPepSearch | MinInt | 50 |
|  | MinMF | 800 |
|  | Hits | 1 |
|  | NIST library | mainlib & replib |

**Supplementary Table 17. Parameters used for feature-based molecular networking.**

| **Step** | **Parameter** | **Value** |
| --- | --- | --- |
| Feature-based-molecular-networking (v28.2) | quantification table source | Mzmine |
|  | precursor ion mass tolerance | 0.02 Da |
|  | fragment ion mass tolerance | 0.02 Da |
|  | min pairs cos | 0.7 |
|  | network topk | 5 |
|  | min matched fragment ions | 6 |
|  | max connected component size | 50 |
|  | library search min matched peaks | 6 |
|  | search analogs | no |
|  | top results to report per query | 1 |
|  | score threshold | 0.7 |
|  | filter precursor window | yes |
|  | filter peaks in 50 Da window | no |
|  | filter library | yes |
|  | normalization | no |
| NAP CCMS2 (v1.2.5) | number of cluster index | 0 |
|  | n first candidates for consensus score | 10 |
|  | accuracy for candidate search | 15 ppm |
|  | acquisition mode | positive/negative |
|  | adduct types | [M+H], [M+Na]/[M-H], [M+FA-H] |
|  | structure databases | COCONUT, GNPS, SUPNAT, NPAtlas, CHEBI, DRUGBANK |
|  | user provided databases | Hop ISDB |
|  | max candidate structures in graph | 1 |
|  | cosine value to subselect | 0.5 |
|  | fusion result for consensus | yes |
|  | workflow type | MZmine |
| Merge Networks Polarity (v22.1) | PPM tolerance | 2 |
|  | RT tolerance | 0.05 |

Detailed parameters used within the feature-based molecular networking workflow on Apollo hop cone developmental non-volatile spectral data.

**Supplementary Table 18. Overview of differential expressed genes after cluster analysis.**

|  | **Entire DEG set** | **DEG Cluster 1** | **DEG Cluster 2** | **DEG Cluster 3** | **DEG Cluster 4** |
| --- | --- | --- | --- | --- | --- |
| **Number of DEGs** | 3612 | 2204 | 816 | 279 | 176 |
| **Homozygous** | 755 | 447 | 194 | 50 | 30 |
| **Heterozygous** | 1981 | 1262 | 380 | 157 | 109 |
| **Het category 1 - Phase 1** | 409 | 243 | 101 | 48 | 36 |
| **Het category 1 - Phase 2** | 412 | 262 | 105 | 45 | 30 |
| **Het category 2** | 1160 | 732 | 174 | 64 | 42 |
| **Het category 3** | 526 | 304 | 90 | 36 | 22 |

To isolate relevant genes of the glandular fraction during hop cone development, differential expression analysis was applied to generated expression data, which yielded in 3612 differentially expressed genes (DEG). Within this set of DEG, 755 and 1981 were found homozygous (hom) and heterozygous (het), respectively. Subsequently, heterozygous genes of the DEG set were further characterized regarding allele specific expression (ASE), which was defined in three categories: Het category 1 - het genes that are solely present in one phase; het category 2 – ortholog het genes that were found present for both phases; het category 3 – ortholog het genes that are significant differentially expressed between the phases (*P <* 0.05) with a TPM > 1 in at least one stage. Numbers of DEG are given for the total set of 3612 genes as well as the corresponding fractions for each of the four DEG clusters after quality control.

**Supplementary Table 19. Results of the chemo-transcriptional correlation analysis.**

| **Compound** | **DEG Cluster 1** | **DEG Cluster 2** | **DEG Cluster 3** | **DEG Cluster 4** |
| --- | --- | --- | --- | --- |
| **VCs** | 7 | 19 | 7 | 15 |
| Monoterpenoid | 3 | 1 | - | - |
| Sesquiterpenoid | 2 | - | - | 4 |
| Fatty acyl | - | 3 | 1 | - |
| Fatty ester | - | 5 | - | 2 |
| Other | 1 | 4 | 2 | 6 |
| Unknown | 1 | 6 | 4 | 3 |
| **NVCs (subnetwork)** | 0 | 23 | 20 | 1 |
| Phenylpropanoids and polyketides | - | 1 | 1 | - |
| Organic oxygen compounds | - | 1 | - | - |
| Lipids and lipid-like molecules | - | 8 | 10 | 1 |
| Organoheterocyclic compounds | - | 9 | 6 | - |
| Other | - | 4 | 3 | - |

Correlation of DEG cluster information to determined metabolic profiles was based on log_2_(*x*+1) transformed gene expression (after HCA quality control) and chemical data, with the latter comprising 45 NVCs subnetworks and 49 VCs. Hereby, a positive correlation was accounted for if a chemical feature was showing a Pearson coefficient above or equal to 0.9 in at least 5% of DEGs of a given cluster. For this, correlation analysis was conducted using the cor() function from the ‘stats’ R package^34^. Chemical annotation is further provided for VC and NVC with total number of corresponding chemical features given.

**Supplementary Table 20. Two-proportions Z-test results for genome-wide ASE counts.**

| **Chr** | **Stage 1** | **Stage 2** | **Stage 3** | **Stage 4** | **Stage 5** | **S1-S5** |
| --- | --- | --- | --- | --- | --- | --- |
| 1 | 0.4849 | 0.5844 | 0.2107 | 0.6509 | 0.2831 | 0.0621 |
| 2 | 0.6704 | 0.6555 | 0.6678 | 0.8123 | 0.6438 | 0.3279 |
| 3 | 0.2876 | 0.1904 | 0.1321 | 0.1352 | 0.0743 | 0.0010 |
| 4 | 0.3418 | 0.3477 | 0.4199 | 0.4681 | 0.5368 | 0.0612 |
| 5 | 0.0766 | 0.0850 | 0.0849 | 0.1495 | 0.1517 | 0.0002 |
| 6 | 0.6823 | 0.9598 | 0.8768 | 0.6458 | 1.0000 | 0.5676 |
| 7 | 0.4654 | 0.4052 | 0.4660 | 0.5424 | 0.3386 | 0.0675 |
| 8 | 1.0000 | 0.8595 | 0.7215 | 0.8561 | 0.9633 | 0.6884 |
| 9 | 0.6524 | 0.6589 | 0.4698 | 0.4321 | 0.5600 | 0.1600 |
| 10 | 0.1268 | 0.0675 | 0.2681 | 0.1499 | 0.1194 | 0.0006 |

Two-proportions Z-tests were conducted on binary counts representing allele specific expression (ASE) within each chromosome (Chr) and phase based on cone development gene expression data comprising five stages (S1-S5). The table shows *P* for each two-proportions Z-test conducted between the phases of each stage as well as combination of all stages (S1-S5). These results indicate that significant (*P* < 0.05) differences in proportions were only found in chromosome 3, 5 and 10 upon combination of ASE counts from all five stages.

**Supplementary references**

1. Grotewold, E. Plant metabolic diversity: a regulatory perspective. *Trends Plant Sci.* **10**, 57–62 (2005).

2. Petrovska, B. B. Historical review of medicinal plants’ usage. *Pharmacogn. Rev.* **6**, 1–5 (2012).

3. Hartmann, T. From waste products to ecochemicals: Fifty years research of plant secondary metabolism. *Phytochemistry* **68**, 2831–2846 (2007).

4. Almaguer, C., Schönberger, C., Gastl, M., Arendt, E. K. & Becker, T. *Humulus lupulus* **-**a story that begs to be told: a review. *J. Inst. Brew.* **120,** 289–314 (2014).

5. Kawamoto, T. & Kawamoto, K. Preparation of thin frozen sections from nonfixed and undecalcified hard tissues using Kawamoto’s film method. In *Skeletal Development and Repair: Methods and Protocols* (ed. Hilton, M. J.) 259–281 (Springer US, New York, 2021).

6. Montini, L. *et al.* Matrix-assisted laser desorption/ionization-mass spectrometry imaging of metabolites during *Sorghum* germination. *Plant Physiol.* **183**, 925–942 (2020).

7. Gericke, O. *et al.* Nerylneryl diphosphate is the precursor of serrulatane, viscidane and cembrane-type diterpenoids in *Eremophila* species. *BMC Plant Biol.* **20**, 91 (2020).

8. Schuurink, R. & Tissier, A. Glandular trichomes: micro-organs with model status? *New Phytol.* **225**, 2251–2266 (2020).

9. Wang, G. *et al.* Terpene biosynthesis in glandular trichomes of hop. *Plant Physiol.* **148**, 1254–1266 (2008).

10. Wang, M. *et al.* Sharing and community curation of mass spectrometry data with Global Natural Products Social Molecular Networking. *Nat. Biotechnol.* **34**, 828–837 (2016).

11. Gericke, O. *et al.* Navigating through chemical space and evolutionary time across the Australian continent in plant genus *Eremophila*. *Plant J.* **108**, 555–578 (2021).

12. da Silva, R. R. *et al.* Propagating annotations of molecular networks using in silico fragmentation. *PLoS Comput. Biol.* **14**, e1006089 (2018).

13. Bocquet, L., Sahpaz, S., Hilbert, J. L., Rambaud, C. & Rivière, C. *Humulus lupulus* L., a very popular beer ingredient and medicinal plant: overview of its phytochemistry, its bioactivity, and its biotechnology. *Phytochem. Rev.* **17**, 1047–1090 (2018).

14. Bohlmann, J., Meyer-Gauen, G. & Croteau, R. Plant terpenoid synthases: molecular biology and phylogenetic analysis. *Proc. Natl. Acad. Sci. U.S.A.* **95**, 4126–4133 (1998).

15. Padgitt-Cobb, L. K., Pitra, N. J., Matthews, P. D., Henning, J. A. & Hendrix, D. A. An improved assembly of the “Cascade” hop (*Humulus lupulus*) genome uncovers signatures of molecular evolution and refines time of divergence estimates for the Cannabaceae family. *Hortic. Res.* **10**, uhac281 (2023).

16. Horáková, L. *et al.* Dynamic patterns of repeats and retrotransposons in the centromeres of *Humulus lupulus* L. *New Phytol.* **247**, 2766–2780 (2025).

17. Velasco, R. *et al.* The genome of the domesticated apple (*Malus × domestica* Borkh.). *Nat. Genet.* **42**, 833–839 (2010).

18. Lovell, J. T. *et al.* GENESPACE tracks regions of interest and gene copy number variation across multiple genomes. *eLife* **11**, e78526 (2022).

19. Jeske, R. D. & Brulotte, J. Hop plant named ‘Apollo’. US Plant Patent USPP20200P3 (2009).

20. Patzak, J. *et al.* Evaluation of genetic variability of wild hops (*Humulus lupulus* L.) in Canada and the Caucasus region by chemical and molecular methods. *Genome* **53**, 545–557 (2010).

21. Haunold, A. & Nickerson, G. B. Registration of USDA 19058M male hop germplasm. *Crop Sci.* **28**, 1036 (1988).

22. Haunold, A., Likens, S. T., Nickerson, G. B., Horner, C. E. & Hampton, R. O. Registration of USDA 63015M male hop germplasm (Reg. No. GP 14). *Crop Sci.* **23**, 600–601 (1983).

23. Patzak, J., Henychová, A. & Matoušek, J. Developmental regulation of lupulin gland-associated genes in aromatic and bitter hops (*Humulus lupulus* L.). *BMC Plant Biol.* **21**, 534 (2021).

24. Ødum, M. T. *et al.* DeepLoc 2.1: multi-label membrane protein type prediction using protein language models. *Nucleic Acids Res.* **52**, W215–W220 (2024).

25. Blum, M. *et al.* InterPro: the protein sequence classification resource in 2025. *Nucleic Acids Res.* **53**, D444–D456 (2025).

26. Altschul, S. F., Gish, W., Miller, W., Myers, E. W. & Lipman, D. J. Basic local alignment search tool. *J. Mol. Biol.* **215**, 403–410 (1990).

27. Li, H. *et al.* A heteromeric membrane-bound prenyltransferase complex from hop catalyzes three sequential aromatic prenylations in the bitter acid pathway. *Plant Physiol.* **167**, 650–659 (2015).

28. Guo, X., Shen, H., Liu, Y. *et al.* Enabling heterologous synthesis of lupulones in the yeast *Saccharomyces cerevisiae*. *Appl. Biochem. Biotechnol.* **188**, 787–797 (2019).

29. U.S. Department of Agriculture (USDA). *Hop cultivar pedigree*. USDA Agricultural Research Service (ARS) (n.d.).

30. Henning, J. A., Townsend, M. S., Gent, D. H., Wiseman, M. S., Walsh, D. B., Groenendale, D. P. & Randazzo, A. M. Registration of high-yielding aroma hop (*Humulus lupulus* L.) cultivar ‘USDA Triumph’. *J. Plant Regist.* **15**, 244–252 (2021).

31. Kenny, S. T. & Zimmermann, C. E. Registration of Olympic hop (Registration No. 14). *Crop Sci.* **24**, 618–619 (1984).

32. Haunold, A., Nickerson, G. B., Gampert, U. & Kenny, S. T. Liberty and Crystal – two new US-developed aroma hops. *J. Am. Soc. Brew. Chem.* **53**, 9–13 (1995).

33. Townsend, M. S. & Henning, J. A. Potential heterotic groups in hop as determined by AFLP analysis. *Crop Sci.* **45**, 1901–1907 (2005).

34. R Core Team. *R: A language and environment for statistical computing*. (R Foundation for Statistical Computing, Vienna, Austria, 2024).
