# Supplementary material for: Extensive variation between chromosomes of North American and European hop
Source: Nat Commun. 2026 May 27;17:4110. doi: 10.1038/s41467-026-72379-8 (PMC13216280; doi:10.1038/s41467-026-72379-8)
Supplement: Supplementary file 3 — Description of Additional Supplementary Files [file 41467_2026_72379_MOESM3_ESM.pdf]

Description of Additional Supplementary Files

File Name: Supplementary Data 1
Description: Statistic of read aligned to the phase2 of Apollo assembly for samples from different bi-parental mapping populations.

File Name: Supplementary Data 2
Description: List of accession used for population genomics study along with the ancestry coefficient identified from admixture analysis.

File Name: Supplementary Data 3
Description: Statistic of read aligned to the phase2 of Apollo assembly for samples used in population genomics study.

File Name: Supplementary Data 4
Description: Quantification of bitter acid content and downy mildew resistance within the ApolloXPubM_740 population.

File Name: Supplementary Data 5
Description: List of RNA-Seq datasets used for gene annotation pipeline.

File Name: Supplementary Data 6
Description: Orthologous gene family expansions and contractions in seven Rosales species.

File Name: Supplementary Data 7
Description: Orthologous gene family expansions and contractions in the Cannabeaceae.

File Name: Supplementary Data 8
Description: Gene ontology enrichment analysis in expanded gene families across Rosales and within the Cannabaceae.

File Name: Supplementary Data 9
Description: Notes and accession numbers for sequences used in Terpene Synthase phylogenetic tree.

File Name: Supplementary Data 10
Description: Notes and accession numbers for sequences used in Aromatic Prenyltranferase phylogenetic tree.

File Name: Supplementary Data 11
Description: Notes and accession numbers for sequences used in Polyketide Synthase phylogenetic tree.

File Name: Supplementary Data 12
Description: Overview on annotation and phase specific expression for Humulus genes shown in Fig 6.

File Name: Supplementary Data 13
Description: Overview on MEP and MVA pathway genes on annotation and expression during cone development in cv. Apollo.

File Name: Supplementary Data 14
Description: Gene content chr08 Eu introgression.

File Name: Supplementary Data 15
Description: Detailed list of volatile reference compounds used in this study.

File Name: Supplementary Data 16
Description: Parameters used for raw LC-MS spectral data processing in MzMine2.

File Name: Supplementary Data 17
Description: Detailed list of non-volatile reference compounds used in this study.
