# Supplementary material for: Extensive variation between chromosomes of North American and European hop
Source: Nat Commun. 2026 May 27;17:4110. doi: 10.1038/s41467-026-72379-8 (PMC13216280; doi:10.1038/s41467-026-72379-8)
Supplement: Supplementary file 21 — Reporting Summary [file 41467_2026_72379_MOESM21_ESM.pdf]

Corresponding author(s): Manuel Spannagl, Alexander Feiner, Ilka Braumann

Last updated by author(s): Mar 26, 2026

Reporting Summary

Nature Portfolio wishes to improve the reproducibility of the work that we publish. This form provides structure for consistency and transparency in reporting. For further information on Nature Portfolio policies, see our Editorial Policies and the Editorial Policy Checklist.

Statistics

For all statistical analyses, confirm that the following items are present in the figure legend, table legend, main text, or Methods section.

n/a	Confirmed	
☐	☒	The exact sample size (*n*) for each experimental group/condition, given as a discrete number and unit of measurement
☐	☒	A statement on whether measurements were taken from distinct samples or whether the same sample was measured repeatedly
☐	☒	The statistical test(s) used AND whether they are one- or two-sided<br>*Only common tests should be described solely by name; describe more complex techniques in the Methods section.*
☐	☒	A description of all covariates tested
☐	☒	A description of any assumptions or corrections, such as tests of normality and adjustment for multiple comparisons
☐	☒	A full description of the statistical parameters including central tendency (e.g. means) or other basic estimates (e.g. regression coefficient) AND variation (e.g. standard deviation) or associated estimates of uncertainty (e.g. confidence intervals)
☐	☒	For null hypothesis testing, the test statistic (e.g. *F*, *t*, *r*) with confidence intervals, effect sizes, degrees of freedom and *P* value noted<br>*Give P values as exact values whenever suitable.*
☒	☐	For Bayesian analysis, information on the choice of priors and Markov chain Monte Carlo settings
☐	☒	For hierarchical and complex designs, identification of the appropriate level for tests and full reporting of outcomes
☐	☒	Estimates of effect sizes (e.g. Cohen's *d*, Pearson's *r*), indicating how they were calculated

Our web collection on statistics for biologists contains articles on many of the points above.

Software and code

Policy information about availability of computer code

Data collection	Raw sequencing data were generated using Illumina (HiSeq 2500 and NovaSeq 6000) and PacBio Sequel II platforms. PacBio HiFi circular consensus sequencing (CCS) reads were generated using SMRTLink software (v9.0.0.92017) and CCS v6.0.0. Raw Illumina sequencing reads were processed using Cutadapt v4.2 and TrimGalore! v0.6.6 to remove adapters and low-quality bases, and Rcorrector v1.0.4 for error correction. Read quality was assessed using FastQC v0.11.9. For RNA-seq datasets, reads were trimmed using Trimmomatic (v0.39.2) additional filtering included removal of rRNA contamination using Bowtie2 v2.4.4 against SILVA-derived databases.Metabolomics raw data from LC–MS and GC–MS platforms were converted to open formats using MSConvert v3.0.2, and spectral matching against reference libraries was performed using MSPepSearch v0.9.4.9. MALDI-MSI data acquisition and visualization were performed using MSiReader v1.01. Chromatographic data acquisition for HPLC analyses was conducted using OpenLab CDS ChemStation v2.3.54.Geographic occurrence data for Humulus species were obtained from GBIF and processed using Python, with mapping performed using Cartopy and Natural Earth datasets.K-mer-based genome size estimation was conducted using KMC v3.2.1 and kmc_tools v3.2.1, followed by modeling in findGSE within the R statistical environment.
Data analysis	Genome assembly and phasing were performed using Hifiasm v0.15.1 and v0.16, with haplotype separation and scaffolding carried out using ALLHiC and the TRITEX pipeline. Hi-C data processing, visualization, and manual curation were conducted using HiC-inspector, Juicebox, and HiCPlotter. Assembly quality and completeness were assessed using BUSCO v5.1.2 and v5.3.2.Sequence alignment and mapping were performed using minimap2 v2.17, v2.22, and v2.24, with downstream processing using samtools v1.15, bcftools v1.11 and v1.15, and NovoSort v3.01.06. Variant calling and filtering were conducted using bcftools v1.15 and VCFtools v0.1.13, while phasing was performed using WhatsHap. Structural variant detection and genome comparison analyses were carried out using RagTag, MUMmer v3.23 and v4.0, SyRI, and visualized using plotsr.Repeat and k-mer analyses were conducted using TandemRepeatFinder v4.09.1, vmatch v2.3.0, tallymer, and KMC v3.2.1. Genome size estimation and additional statistical analyses were performed in R.Gene annotation integrated homology-based, transcriptome-based, and ab initio approaches using GenomeThreader v1.7.1, STAR v2.7.8a, StringTie2 v2.1.5, TransDecoder v3.0.0, BLAST+

v2.13.0, HMMER v3.3.2 (hmmscan), gffread v0.12.1, AUGUSTUS v3.4.0, EVidenceModeler (EVM), and PASA v2.4.1, followed by refinement using Mikado. Additional alignment and consolidation steps used BLAT v34, Exonerate v2.4, and GMAP. Functional annotation was performed using Mercator.Transposable elements were annotated using EDTA, RepeatModeler2, and RepeatMasker. Orthology inference and gene family evolution analyses were performed using OrthoFinder and CAFE5, with divergence time estimates obtained from TimeTree and functional annotation supported by eggNOG v2.1.12 and topGO v2.44.0.Synteny and comparative genomics analyses were conducted usingn MCScanX and GENESPACE v1.3.1. Phylogenetic analyses were performed using MEGA X, MAFFT, BMGE, and FastTree, with visualization using iTOL and additional workflows via NGPhylogeny.fr.Population genomics and GWAS analyses were conducted using GEMMA v0.98.5, GAPIT v3 with the BLINK model, ADMIXTURE v1.3.0, and SNPRelate v1.24.0 (including FastPCA implementation). Visualization of association results was performed using qqman.Transcriptomic analyses were carried out using Salmon v1.9.0, DESeq2 v1.36.0, and edgeR v3.42.4, with clustering and statistical analyses performed in R using packages including amap, cluster, dynamicTreeCut, factoextra, and ggplot2. Allele-specific expression and statistical testing were performed using functions from the stats package and visualized using UpSetR.Metabolomics data processing and feature detection were conducted using MZmine2 v2.5.3, with batch correction using TIGERr v1.0.0. Molecular networking and compound annotation were performed using the GNPS platform, including Feature-Based Molecular Networking (FBMN), Network Annotation Propagation (NAP), MolNetEnhancer v22, and in silico fragmentation tools (Fusion, Consensus, and MetFrag). Networks were visualized using Cytoscape v3.8.2.All statistical analyses, data integration, and visualization were performed in the R statistical environment. The code used for metabolomics data processing and analysis is available at GitHub (https://github.com/carlsberglaboratorium-publications/apollo-cone-development-metabolomics) and archived at Zenodo (https://doi.org/10.5281/zenodo.19134880).

For manuscripts utilizing custom algorithms or software that are central to the research but not yet described in published literature, software must be made available to editors and reviewers. We strongly encourage code deposition in a community repository (e.g. GitHub). See the Nature Portfolio guidelines for submitting code & software for further information.

Data

Policy information about availability of data

All manuscripts must include a data availability statement. This statement should provide the following information, where applicable:

- Accession codes, unique identifiers, or web links for publicly available datasets
- A description of any restrictions on data availability
- For clinical datasets or third party data, please ensure that the statement adheres to our policy

The sequencing data generated in this study have been deposited in the European Nucleotide Archive (ENA) database under accession codes PRJEB64593 (scaffold sequence used for training AUGUSTUS gene annotations, https://www.ebi.ac.uk/ena/browser/view/PRJEB64593), PRJEB64169 (contig sequences and AGP file of the haploid cv Apollo assembly, https://www.ebi.ac.uk/ena/browser/view/PRJEB64169), PRJEB63995 (CCS, RNA-Seq, iso-seq and Hi-C reads used for the generation and annotation of the phased cv. Apollo assembly, https://www.ebi.ac.uk/ena/browser/view/PRJEB63995), PRJEB64122 (RNA-Seq data from the developing Apollo cone, https://www.ebi.ac.uk/ena/browser/view/PRJEB64122), PRJNA1082089 (phased assembly of hop cv Apollo, https://www.ebi.ac.uk/ena/browser/view/PRJNA1082089), PRJEB63565 (linkage mapping Apollo × PubM_740 population, https://www.ebi.ac.uk/ena/browser/view/PRJEB63565), PRJEB63534 (linkage mapping Cascade × HL-19 population, https://www.ebi.ac.uk/ena/browser/view/PRJEB63534), PRJEB63136 (GBS data used for population genomic study, https://www.ebi.ac.uk/ena/browser/view/PRJEB63136).
The annotation of the phased cv. Apollo assembly has been deposited to Zenodo under https://doi.org/10.5281/zenodo.18787309. All input and results for the syntenic and orthologous framework for Humulus and Cannabis were deposited to Zeonodo under https://doi.org/10.5281/zenodo.18802928.
The Raw DHS-GC–MS and LC-qTof-MS/MS spectral data generated on Apollo hop cone development, control and blank samples are available at the MassIVE repository database under ID MSV000095961. All derived molecular networking jobs can be publicly accessed at following links: https://gnps.ucsd.edu/ProteoSAFe/status.jsp?task=0f83c7cfad91444e855c1987f161e854 (FBMN job for positive ionization), https://gnps.ucsd.edu/ProteoSAFe/status.jsp?task=19aa55bc1f564681b104ddd1b4858a82 (FBMN job for negative ionization), https://proteomics2.ucsd.edu/ProteoSAFe/status.jsp?task=025a463a22c54b2da226cf9ebe5c031a (NAP job for positive ionization), https://proteomics2.ucsd.edu/ProteoSAFe/status.jsp?task=7ff6e3f7657240c88bfc11f8e3b360fd (NAP job for negative ionization), https://gnps.ucsd.edu/ProteoSAFe/status.jsp?task=48dd84680d3c4e04b6f6b82e407157cd (MolNetEnhancer job for positive ionization), https://gnps.ucsd.edu/ProteoSAFe/status.jsp?task=bf995243b6e94a49ab702541dc26e1ce (MolNetEnhancer job for negative ionization), https://gnps.ucsd.edu/ProteoSAFe/status.jsp?task=ed53ff8a61af457f8c9bf4de87153d89 (Merged ionization network).

Source data are provided with this paper. Source data for the following items have been deposititeddeposited at Zenodo and are available through the respective links: Fig. 1b (https://doi.org/10.5281/zenodo.17424332), Fig. 2b (https://doi.org/10.5281/zenodo.17941436), Fig. 3a (https://doi.org/10.5281/zenodo.18802928), Fig. 4 (https://doi.org/10.5281/zenodo.17942432), Fig. 5d (https://doi.org/10.5281/zenodo.17942758), Fig. 6c (https://doi.org/10.5281/zenodo.19065883), Fig. 7a (https://doi.org/10.5281/zenodo.17942092), Fig. 7b (https://doi.org/10.5281/zenodo.17942366), Supplementary Fig. 20a, b and c (https://doi.org/10.5281/zenodo.17943184), Supplementary Fig. 21 (https://doi.org/10.5281/zenodo.17943272), Supplementary Fig. 24 (https://doi.org/10.5281/zenodo.19088367), Supplementary Fig. 25 (https://doi.org/10.5281/zenodo.17943626 and https://doi.org/10.5281/zenodo.17943656), Supplementary Fig. 28 (https://doi.org/10.5281/zenodo.19065040) Supplementary Fig. 30, and Supplementary Fig. 32 (https://doi.org/10.5281/zenodo.19065806) and Supplementary Data 12 (https://doi.org/10.5281/zenodo.19128409).

The following previously published data sets were used in the current study:
Occurrence datasets from the Global Biodiversity Information Facility (GBIF) for generation of Fig. 1b: GBIF.org https://doi.org/10.15468/dl.w53rkc , https://doi.org/10.15468/dl.cr37wu, https://doi.org/10.15468/dl.6ye7ug , https://doi.org/10.15468/dl.8jnu9g , https://doi.org/10.15468/dl.mn7b6d , and https://doi.org/10.15468/dl.rzwt9h)
Genome assemblies for Humulus cv.Cascade (Supplementary Fig. 4, https://hopbase.org) and for Cannabis sativa cv. Cs10 (Fig.3b,c; https://www.ncbi.nlm.nih.gov/search/all/?term=GCA_900626175.2).

Gene annotation files for Humulus cvs. Saaz (Fig 3a https://plantgarden.jp/en/list/t3486/genome/t3486.G004), Cascade (Fig.3a, Supplementary Fig. 20d https://hopbase.org), and drHumLupu1.1 (Supplementary Fig. 21 https://www.ncbi.nlm.nih.gov/datasets/genome/GCF_963169125.1/) and Cannabis sativa cvs. Pink Pepper (Fig. 3a https://www.ncbi.nlm.nih.gov/search/all/?term=GCA_029168945.1) and Cs10 (Supplementary Fig.s 4b, Fig. 5, 20d, 23 https://ftp.ncbi.nlm.nih.gov/genomes/all/GCF/900/626/175/GCF_900626175.2_cs10/), Parasponia andersonii (Fig. 5 https://www.ncbi.nlm.nih.gov/datasets/genome/GCA_002914805.1/), Malus domestica (Supplementary Fig.s 20d, 23 https://ftp.ncbi.nlm.nih.gov/genomes/all/GCF/002/114/115/GCF_002114115.1_ASM211411v1/), Prunus persica (Supplementary Fig.s 20d, 23 https://ftp.ncbi.nlm.nih.gov/genomes/all/GCA/000/346/465/GCA_000346465.2_Prunus_persica_NCBIv2/), Vitis vinifera (Supplementary Fig.s 20d, 23 https://ftp.ncbi.nlm.nih.gov/genomes/all/GCA/000/003/745/GCA_000003745.2_12X), Trema orientale (Fig. 5, Supplementary Fig. 23 https://ftp.ncbi.nlm.nih.gov/genomes/all/GCA/002/914/845/GCA_002914845.1_TorRG33x02_asm01/), Morus notabilis (Supplementary Fig. 23 https://

ftp.ncbi.nlm.nih.gov/genomes/all/GCF/000/414/095/GCF_000414095.1_ASM41409v2/) and Ziziphus jujuba (Supplementary Fig.s 20d, 23 https://ftp.ncbi.nlm.nih.gov/genomes/all/GCF/000/826/755/GCF_000826755.1_ZizJuj_1.1/).
Raw sequence data for the Zenith × USDA21058M biparental population (Supplementary Fig. 12b, https://www.ncbi.nlm.nih.gov/bioproject/?term=PRJNA906612).

Research involving human participants, their data, or biological material

Policy information about studies with human participants or human data. See also policy information about sex, gender (identity/presentation), and sexual orientation and race, ethnicity and racism.

Reporting on sex and gender	no research on humans involved
Reporting on race, ethnicity, or other socially relevant groupings	no research on humans involved
Population characteristics	no research on humans involved
Recruitment	no research on human involved
Ethics oversight	no research on humans involved

Note that full information on the approval of the study protocol must also be provided in the manuscript.

Field-specific reporting

Please select the one below that is the best fit for your research. If you are not sure, read the appropriate sections before making your selection.

☒ Life sciences ☐ Behavioural & social sciences ☐ Ecological, evolutionary & environmental sciences

For a reference copy of the document with all sections, see nature.com/documents/nr-reporting-summary-flat.pdf

Life sciences study design

All studies must disclose on these points even when the disclosure is negative.

Sample size	No formal a priori sample size calculation was performed. Sample sizes were determined by the availability of biological material, the design of the respective experiments, and standard practice for the methods used. For genetic analyses, the full available mapping populations were used (182 individuals from the Cascade × HL19 F1 population and 184 individuals from the Apollo × PubM740 F1 population). For female-specific traits, only female individuals from the Apollo × PubM740 population were included. For the diversity panel, all available accessions were analyzed. For transcriptomic, metabolomic, chemical, and PCR-based experiments, sample sizes were chosen based on the availability of independent biological material and were considered appropriate to assess reproducibility; details of biological replication are provided under "Replication".
Data exclusions	no data was excluded from the analysis
Replication	All attempts of replication were successful. RNAseq experiments (PRJEB64122 – RNA-Seq from Apollo cone developmental stages and PRJEB91339 – Transcriptomes from cones of Apollo × PubM740 hop hybrids): The samples represent biological triplicates. The HPLC data for the females of the ApolloXPubM_740 population were generated from two crop years (2018 and 2021), each with two distinct sample time points of the same individuals. Within each sample time point, two biological replicates were taken. Metabolomics data of the developing cones were generated from biological triplicates. PCR experiments were successfully replicated as described in the respective figure legends.
Randomization	Samples were not randomly allocated into experimental groups because this study did not involve investigator-assigned treatment groups. Rather, samples were analyzed according to inherent or predefined biological categories, such as genotype, sex, developmental stage, tissue type, crop year, and population. Thus, randomization was not relevant to the study design.
Blinding	Blinding was not applied in this study because samples were defined by objective biological characteristics (for example genotype, sex, developmental stage, tissue type, and crop year), rather than by investigator-assigned treatment groups. Data collection and analysis were based on standardized molecular and chemical workflows, including sequencing-based assays, PCR-based validation, and HPLC/metabolomics measurements, with outcomes determined by instrument readouts and bioinformatic pipelines rather than subjective investigator scoring.

Reporting for specific materials, systems and methods

We require information from authors about some types of materials, experimental systems and methods used in many studies. Here, indicate whether each material, system or method listed is relevant to your study. If you are not sure if a list item applies to your research, read the appropriate section before selecting a response.

Materials & experimental systems

n/a	Involved in the study	
☒	☐	Antibodies
☒	☐	Eukaryotic cell lines
☒	☐	Palaeontology and archaeology
☒	☐	Animals and other organisms
☒	☐	Clinical data
☒	☐	Dual use research of concern
☐	☒	Plants

Methods

n/a	Involved in the study	
☒	☐	ChIP-seq
☒	☐	Flow cytometry
☒	☐	MRI-based neuroimaging

Dual use research of concern

Policy information about dual use research of concern

Hazards

Could the accidental, deliberate or reckless misuse of agents or technologies generated in the work, or the application of information presented in the manuscript, pose a threat to:

No	Yes	
☒	☐	Public health
☒	☐	National security
☒	☐	Crops and/or livestock
☒	☐	Ecosystems
☒	☐	Any other significant area

Experiments of concern

Does the work involve any of these experiments of concern:

No	Yes	
☒	☐	Demonstrate how to render a vaccine ineffective
☒	☐	Confer resistance to therapeutically useful antibiotics or antiviral agents
☒	☐	Enhance the virulence of a pathogen or render a nonpathogen virulent
☒	☐	Increase transmissibility of a pathogen
☒	☐	Alter the host range of a pathogen
☒	☐	Enable evasion of diagnostic/detection modalities
☒	☐	Enable the weaponization of a biological agent or toxin
☒	☐	Any other potentially harmful combination of experiments and agents

Plants

Seed stocks	No plant was specially sourced for this study. As Hop is a perennial species that is not propagated from seed stocks, all plants are maintained in the contributor collections. The origin of all plants that are neither breeding lines nor non-commercial cultivars is summarzed in Supplementary Data 2 of the supplemental tables.
Novel plant genotypes	Non-novel plant genotypes were generated in this study.
Authentication	na
